# Potent triazine-based dehydrocondensing reagents substituted by an amido group

**DOI:** 10.3762/bjoc.12.179

**Published:** 2016-08-24

**Authors:** Munetaka Kunishima, Daiki Kato, Nobu Kimura, Masanori Kitamura, Kohei Yamada, Kazuhito Hioki

**Affiliations:** 1 Faculty of Pharmaceutical Sciences, Institute of Medical, Pharmaceutical, and Health Sciences, Kanazawa University, Kakuma-machi, Kanazawa 920-1192, Japan; 2 Faculty of Pharmaceutical Sciences, Kobe Gakuin University, 1-1-3 Minatojima Chuo-ku, Kobe 655-8586, Japan

**Keywords:** amide-forming reactions, amido substituents, dehydrocondensing reactions, Fischer-type esterification, triazines

## Abstract

This study describes the synthesis of triazine-based dehydrocondensing reagents substituted by amido substituents and demonstrates their efficiency for dehydrocondensing reactions in MeOH and THF. *N*-Phenylbenzamido-substituted chlorotriazine is readily converted to a stable, non-hygroscopic triazinylammonium-based dehydrocondensing reagent that is superior to 4-(4,6-dimethoxy-1,3,5-triazin-2-yl)-4-methylmorpholinium chloride (DMT-MM) in terms of its reactivity in dehydrocondensing reactions.

## Introduction

**Scheme 1:**
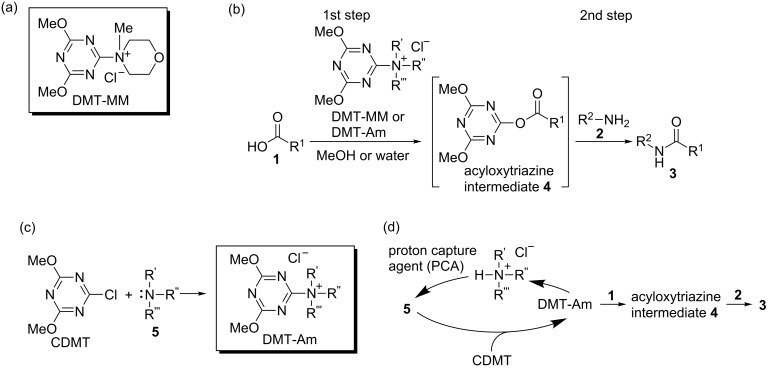
Dehydrocondensing reactions using DMT-MM or DMT-Am, and a catalytic amide-forming reaction.

We previously reported that dehydrocondensing reactions between carboxylic acids **1** and amines **2** to give amides **3** efficiently proceed in water or alcohols in the presence of the dehydrocondensing reagent, 4-(4,6-dimethoxy-1,3,5-triazin-2-yl)-4-methylmorpholinium chloride (DMT-MM) (Scheme 1a,b) [1–5]. The mechanism of the reaction shown in Scheme 1b involves the acyloxytriazine intermediate **4**, which has appropriate reactivity and sufficient stability to hydrolysis or alcoholysis. This feature enables the use of DMT-MM for dehydrocondensing reactions in water or alcohols to give amides without the recovery of hydrolyzed carboxylic acids and the formation of corresponding esters. DMT-MM is quantitatively synthesized from 2-chloro-4,6-dimethoxy-1,3,5-triazine (CDMT) and *N*-methylmorpholine (NMM). Related triazinylammonium salts (DMT-Am) can be prepared from other tertiary amines **5** instead of NMM (Scheme 1c) [6].

Since the direct reaction of carboxylates with CDMT is very slow, the formation of DMT-Am is essential for the conversion of carboxylates into **4** [7]. Therefore, we developed catalytic amide-forming reactions in water using triazinylammonium salts (DMT-Am) generated in situ from CDMT and a catalytic amount of tertiary amines **5** (Scheme 1d). Most notably, by introducing various functional groups into **5**, it was possible to develop a cyclodextrin-based artificial acyltransferase [7], a crown-ether-based cyclotransferase [8], membrane fusion of small unilamellar vesicles to form giant unilamellar vesicles [9], modular methods for the affinity labeling of targeting proteins [10–12], and reaction acceleration on micelle interfaces [13–14]. Thus, various types of molecular recognitions of carboxylic acids were affected by fuctionalized DMT-Am. However, once the reaction with **1** takes place, the functionalized tertiary amines **5** liberated, no longer affect the following reaction (Scheme 1d). As a consequence, the resulting acyloxyatriazine intermediate **4**, which is the same intermediate in Scheme 1b, basically shows the same reactivity for aminolysis and alcoholysis regardless of which tertiary amine **5** was used (Scheme 1b,d). Therefore, it is reasonable to modify the activity of the 1,3,5-triazine ring that is involved in both steps shown in Scheme 1b by introducing another substituent in place of the methoxy groups.

To date, several triazine derivatives possessing phenoxy [15–16], 2,2,2-trifluoroethoxy [17], or *N*-ethylamino groups [18] in place of the methoxy groups have been prepared, and the reactivity of these compounds in amide-forming reactions has been examined. The reactivity of triazine was found to slightly increase following the introduction of electron-withdrawing phenoxy or 2,2,2-trifluoroethoxy groups and decrease upon the inclusion of an electron-donating *N*-ethylamino group. This result may indicate that the tuning of the π-electron density within the triazine ring is important in either enhancing or suppressing its reactivity. To find a novel triazine-based dehydrocondensing reagent, we are focusing on the effect of substituents on the triazine ring. In this paper, we report the synthesis and reactivity of triazine-based dehydrocondensing reagents that have an amido group in place of the methoxy groups of CDMT or DMT-MM.

## Results and Discussion

**Figure 1:**
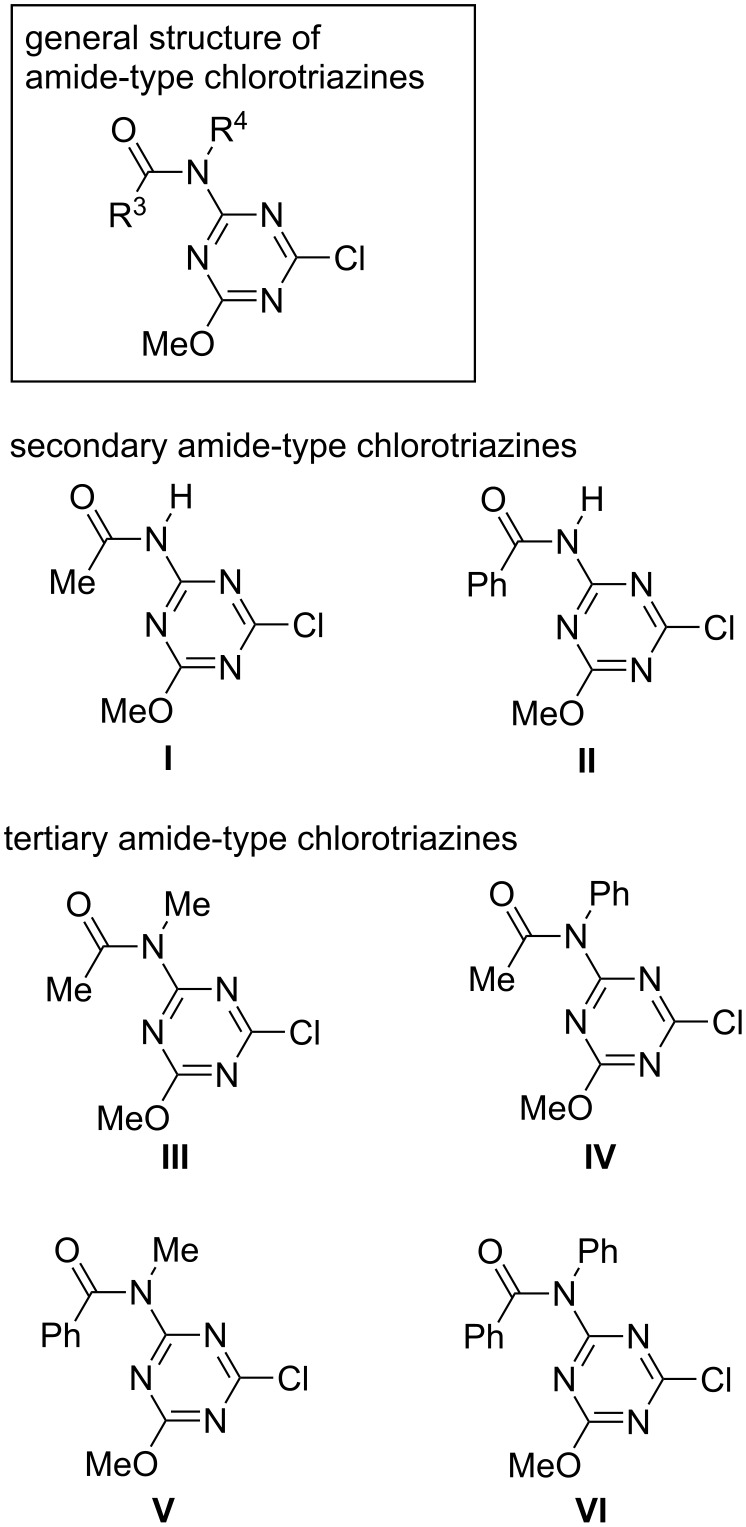
Structures of amido-substituted chlorotriazines.

We decided to use an amido group as a substituent on the triazine ring instead of a methoxy group based on Hammett substituent constants, which indicate that an acetamido group is a more electron-withdrawing group (σ_m_ = 0.31 for –NMeCOMe, 0.21 for –NHCOMe, and 0.12 for –OMe [19]). In addition, we expected that the appropriate substitution pattern of amido groups (R^3^ and R^4^ in Figure 1) would enable the precise control of the reactivity of triazines in dehydrocondensing reactions. We therefore synthesized various secondary or tertiary amide-type chlorotriazines (**I**–**VI**) by the substitution of only one methoxy group of CDMT because it is straightforward to evaluate the effect of a single amido substituent. For the design and synthesis of **I**–**VI**, acetamido and benzamido groups were selected as the amido substituents (R^3^ = Me or Ph), and the methyl, phenyl, or hydrogen substituent was placed at R^4^.

**Scheme 2:**
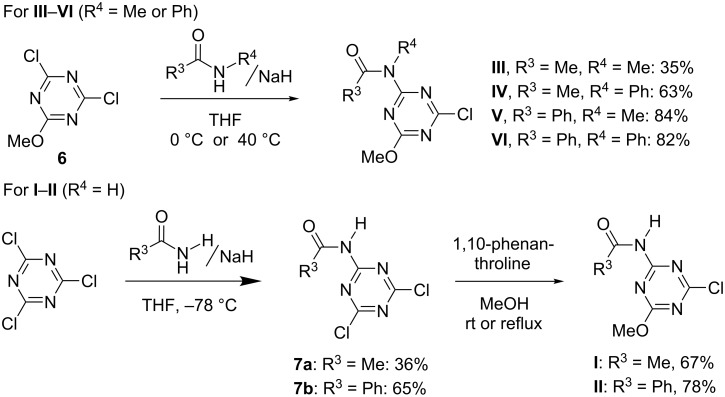
Synthesis of amido-substituted chlorotriazines.

Tertiary amide-type compounds **III**–**VI** were prepared in one step from the well-known commercially available 2,4-dichloro-6-methoxy-1,3,5-triazine (**6**) [20] (Scheme 2). In contrast, attempts to prepare the secondary amide-type **I** and **II** using the same method resulted in low yields mainly because of the substitution of the methoxy group of **6** by amido anions (Supporting Information File 1). Thus, these compounds were successfully synthesized using an alternative method starting from cyanuric chloride via an intermediate **7** (Scheme 2).

Single-crystal X-ray structure analysis, two-dimensional NMR spectroscopy, or derivatization of **I**–**VI** was performed to unambiguously determine their structures, i.e., whether the *N*- or *O*-substituted derivative was prepared from the amido anion (Supporting Information File 1).

To evaluate the reactivity of these chlorotriazines in amide-forming reactions, one-pot reactions were performed (Table 1a), i.e., the chlorotriazines were added to a mixture of carboxylic acid **1a**, amine **2a**, and NMM in MeOH or THF at room temperature. The amide-forming reaction in the absence of NMM did not proceed, meaning that triazinylmorpholinium salts are the actual reactive species (Supporting Information File 1). To permit an accurate comparison between the chlorotriazines, all amide-forming reactions presented in Table 1 were conducted for 3 h.

**Table 1:** Amide-forming reactions using chlorotriazines (**I**–**VI**) or triazinylammonium salts (**VII**–**X**).

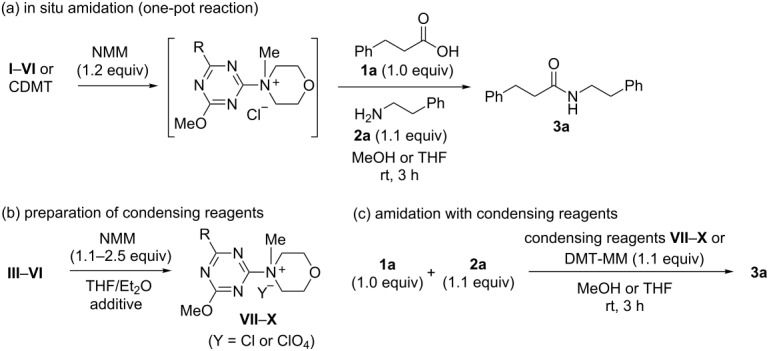									

chlorotriazine	(a) in situ amidation			(b) preparation of condensing reagents (**VII**–**X**)				(c) amidation using **VII**–**X**	
	yield (%)							yield (%)	
		
	in MeOH	in THF			additive	yield (%)		in MeOH	in THF

CDMT	88	81		DMT-MM	–	100		98	84

**I**	70	55		–^a^	–^a^	–^a^		–^a^	–^a^
**II**	86	80		–^a^	–^a^	–^a^		–^a^	–^a^
**III**	95	60		**VII** (Y =ClO_4_)^b^	LiClO_4_ (1.1 equiv)	98		75	50
**IV**	90	73		**VIII** (Y = ClO_4_)^b^	LiClO_4_ (1.1 equiv)	91		88	84
**V**	91	72		**IX** (Y = Cl)^b^	–	90		75	81
**VI**	93	79		**X** (Y = Cl)^b^	–	80		95	89

^a^The reaction was not conducted. ^b^The counter anions were shown in parentheses.

Compared with CDMT, low yields of the amide **3a** were obtained using the secondary amide-type **I** and **II**, possibly because of the low solubilities of **I** and **II** in the reaction solvents owing to the acidic proton of the secondary amide. When using **III**–**VI**, which were soluble in reaction solvents, the yields of **3a** in both MeOH and THF, particularly the high yield of **3a** obtained using benzamido-substituted **VI**, were comparable with those obtained using CDMT.

From these results, dehydrocondensing reagents (**VII**–**X**, Table 1) were prepared from **III**–**VI** and NMM (Table 1b). Although benzamido-substituted **IX** and **X** could be readily synthesized, acetamido-substituted triazines were hygroscopic, and therefore, **VII** and **VIII** were prepared as stable, non-hygroscopic reagents by counterion exchange in the presence of LiClO_4_ [6].

Amide-forming reactions using the dehydrocondensing reagents **VII**–**X** were then examined (Table 1c). While **X** afforded a good yield (95% in MeOH and 89% in THF) of the amide **3a** in both solvents, slightly low yields (75–88% in MeOH and 50–84% in THF) for **VII** and **VIII** were obtained, possibly because of the low solubilities of these reagents in the investigated solvents (perchlorate salts have generally low solubilities).

We then analyzed the NMR spectra of crude mixtures obtained from the reactions using **III** (THF solvent, Table 1a) or **VII** (THF solvent, Table 1c) and identified *N*-acetyl-2-phenylethylamine in 29% and 25% yields, respectively. This byproduct was formed by the aminolysis of the *N*-methylacetamido group on the triazine ring by **2a**. A similar phenomenon was observed for **IV** or **VIII** in THF. Notably, such a byproduct was not obtained in the case of **V**, **IX, VI**, and **X**. In MeOH, no such byproduct resulting from aminolysis was observed in all the cases.

We employed **X** to further study the synthesis of various amides with a particular focus on the stability and non-hygroscopic property of the reagent (Table 2). In most cases, **X** exhibited similar reactivity in MeOH compared with DMT-MM, and the amides **3** were obtained with negligible formation of the corresponding methyl ester **8** (Table 2, entries 1, 5, 7, and 9). In the case of the sterically hindered secondary amine **2c**, methyl ester **8** was obtained as a byproduct in 31% and 24% yields for **X** and DMT-MM (Table 2, entry 3), respectively. In contrast, the yields obtained in THF using **X** were superior to those obtained using DMT-MM (Table 2, entries 4, 6, 8, and 10), especially for the poorly nucleophilic aniline **2b** (Table 2, entry 2).

**Table 2:** Substrate scope of carboxylic acids **1** and amines **2**.

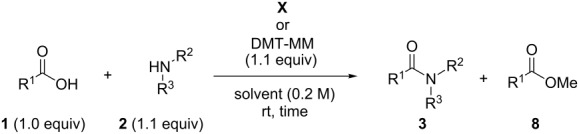											

					**X**				DMT-MM		
			
					time (h)	yield (%)^a^			time (h)	yield (%)^a^	
			
entry	carboxylic acid	amine	amide	solvent		**3**	**8**			**3**	**8**

1	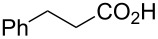 **1a**	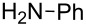 **2b**	**3b**	MeOH	6	80	(1)		24	73	(0)
2				THF	6	75	–		24	16	–
		
3	**1a**	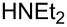 **2c**	**3c**	MeOH	6	65	(31)		9	56	24
4				THF	4	85	–		4	68^b^	–
		
5	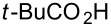 **1b**	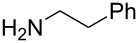 **2a**	**3d**	MeOH	6	(87)	(3)		6	(89)	(0)
6				THF	3	(90)	–		3	(86)	–
		
7	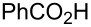 **1c**	**2a**	**3e**	MeOH	4	97	(8)		3	90	(1)
8				THF	4	95	–		4	81^b^	–
		
9	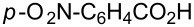 **1d**	**2a**	**3f**	MeOH	4	91	(2)		3	86	(4)
10				THF	4	96	–		3	82^b^	–

^a^Isolated yields. ^1^H NMR yields are given in parentheses. ^b^From reference [2].

**Figure 2:**
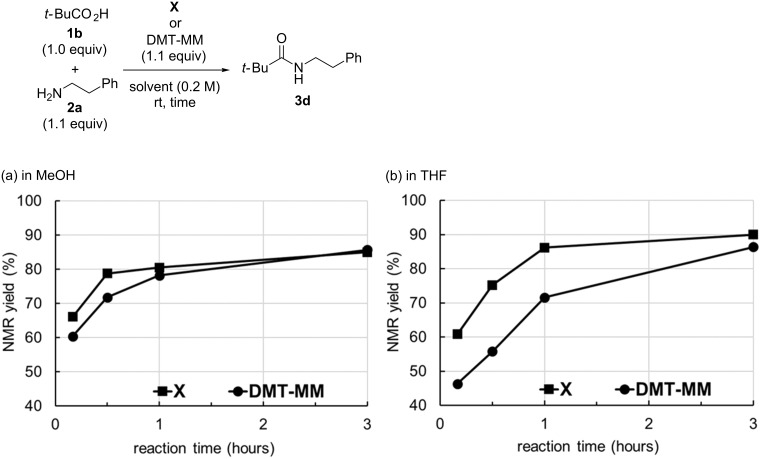
Time courses of the amide-forming reactions.

To elucidate the reactivities of **X** and DMT-MM, time courses of amide-forming reactions using sterically hindered pivalic acid (**1b**) and 2-phenylethylamine (**2a**) in MeOH and THF were investigated by ^1^H NMR spectroscopy (Figure 2a,b, respectively). The steric hindrance of **1b** was suitable to investigate the reaction rates for **X** and DMT-MM. Although the final yields of the amide were almost the same for both triazines, the rate of the reaction with **X** was faster than that with DMT-MM, particularly in THF.

**Figure 3:**
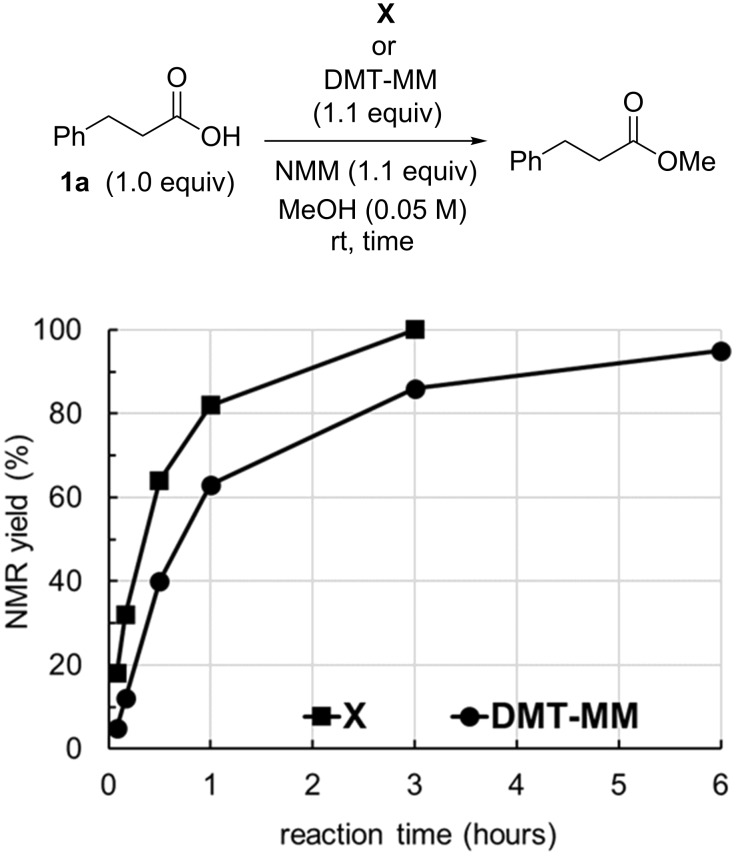
Time courses of the basic Fischer-type esterification.

Similarly, time courses of the basic Fischer-type esterification of 3-phenylpropanoic acid (**1a**) in neat MeOH were studied (Figure 3). Although **X** was shown to exist as a dihydrate by elemental analysis and single-crystal X-ray structure analysis, the yield of methyl 3-phenylpropanoate was quantitative. The rate of esterification with **X** was faster than that with DMT-MM.

## Conclusion

We prepared triazine-based dehydrocondensing reagents bearing amido substituents and demonstrated their efficiency for their use in dehydrocondensing reactions in both MeOH and THF. The tertiary amide-type chlorotriazines **III**–**VI** gave better yields of the product **3a** compared with the secondary amide-type chlorotriazines **I** and **II**, possibly because of the reduced solubilities of **I** and **II** in the reaction solvents. **VI** is readily converted to a triazinylammonium-based dehydrocondensing reagent as a chloride salt (**X**), which is stable and non-hygroscopic. The dehydrocondensing reagent **X** is superior to DMT-MM in terms of reactivity in dehydrocondensing reactions. Although DMT-MM is the appropriate reagent for dehydrocondensing reactions in large-scale synthesis, **X** have advantage for dehydrocondensing reactions in aprotic solvents such as THF, especially when low nucleophilic starting materials such as aniline were used. This study contributes to the development of novel functionalized triazine-based dehydrocondensing reagents that are effective for aminolysis or alcoholysis shown in the second step of Scheme 1b.

## Supporting Information

General information, synthesis of triazines **I**–**X** and structure determination, spectral data of amides **3**, attempted synthesis of **I** from **6**, amide-forming reaction with **VI** in the absence of NMM, and copies of ^1^H and ^13^C NMR spectra of unknown compounds are given.

File 1 Experimental and characterization data.
