# Supplementary material for: Potent triazine-based dehydrocondensing reagents substituted by an amido group
Source: Beilstein J Org Chem. 2016 Aug 24;12:1897–903. doi: 10.3762/bjoc.12.179 (PMC5082469; doi:10.3762/bjoc.12.179)
Supplement: File 1 — Experimental and characterization data. [file Beilstein_J_Org_Chem-12-1897-s001.pdf]

Supporting Information

for

Potent triazine-based dehydrocondensing reagents substituted by an amido group

Munetaka Kunishima*[1], Daiki Kato[1], Nobu Kimura[1], Masanori Kitamura[1], Kohei Yamada[1] and Kazuhito Hioki[2]

Address: [1]Faculty of Pharmaceutical Sciences, Institute of Medical, Pharmaceutical, and Health Sciences, Kanazawa University, Kakuma-machi, Kanazawa 920-1192, Japan and [2]Faculty of Pharmaceutical Sciences, Kobe Gakuin University, 1-1-3 Minatojima Chuo-ku, Kobe 655-8586, Japan

Email: Munetaka Kunishima* - kunisima@p.kanazawa-u.ac.jp

* Corresponding author

Experimental and characterization data

Contents

General information ··· S3
Synthesis of chlorotriazines **I**–**VI** ··· S3–S6
Synthesis of triazinylammonium salts **VII**–**X** ··· S6–S8
Spectral data of amides **3** ··· S8–S9
HMBC spectra of **III** and **V** ··· S10–S11
Single crystal X-ray structure analysis of **II**, **VIII**, and **X** ··· S12–S14
Structure determination of **I** by methylation ··· S15
Attempted synthesis of **I** from **6** ··· S16
Amide-forming reaction with **VI** in the absence of NMM ··· S17
Copies of ^{1}H and ^{13}C NMR spectra ··· S18–S36
References ··· S37

General information

Chemical shifts for ^{1}H NMR spectra are reported as δ values relative to tetramethylsilane as an internal standard for chloroform-*d*, DMSO-d_6, and methanol-d_4. Coupling constants are in hertz (Hz). The following abbreviations are used for spin multiplicity: s = singlet, d = doublet, t = triplet, q = quartet, m = multiplet, and br = broad. NMR yields were determined by quantitative NMR spectroscopy using coumarin, 6-methylcoumarin, or *p*-nitrotoluene as an internal standard. Chemical shifts for ^{13}C NMR are reported as δ values relative to the center lines of the signals at 77.16 ppm corresponding to chloroform-*d*, 39.52 ppm corresponding to DMSO-d_6, or 49.00 ppm corresponding to methanol-d_4. Flash chromatography separations were performed on KANTO CHEMICAL Silica Gel 60 N (spherical, neutral, 40–100 mesh). All reactions sensitive to oxygen or moisture were conducted under a nitrogen atmosphere. *N*-Methylacetamide, acetamide, and benzamide were purchased from Tokyo Chemical Industry Co., LTD. *N*-Phenylacetamide was prepared from acetyl chloride and aniline. *N*-Methylbenzamide, and *N*-phenylbenzamide were prepared using DMT-MM in methanol from benzamide and methylamine, or benzamide and aniline.

2-Chloro-4-methoxy-6-(N-methylacetamido)-1,3,5-triazine (III)

N-Methylacetamide (1.50 g, 20.5 mmol) in THF (10 mL) was added to a suspension of sodium hydride (1.39 g, 34.9 mmol, 60% dispersion in oil) in THF (10 mL) at 0 °C. After stirring for 2 h at 40 °C, the reaction mixture was cooled to 0 °C. 2,4-Dichloro-6-methoxy-1,3,5-triazine (**6**) (3.69 g, 20.5 mmol) was added to the suspension, and the mixture was stirred at 40 °C for two days. The reaction mixture was quenched with sat. aq. NH_4Cl at 0 °C, extracted with AcOEt. The organic layer was washed with brine, dried over Na_2SO_4, filtered, and concentrated under reduced pressure. The residue was purified by column chromatography (hexane/AcOEt 9:1) to afford 2-chloro-4-methoxy-6-(*N*-methylacetamido)-1,3,5-triazine (**III**) (1.53 g, 35%) as a white powder. M.p. 35–36 °C; ^{1}H NMR (400 MHz, $CDCl_3$, TMS): δ=2.70 (s, 3H), 3.47 (s, 3H), 4.07 (s, 3H); ^{13}C NMR (100 MHz, $CDCl_3$): δ=28.31, 32.56, 56.11, 167.94, 171.26, 171.57, 173.16; IR (KBr): 3016, 2952, 1707, 1558, 1362, 1223, 1055, 993 cm^{-1}; HRMS (EI–sector): *m*/*z* [M]$^+$ calcd for $C_7H_9ClN_4O_2$: 216.0414; found: 216.0416; elemental analysis calcd (%) for $C_7H_9ClN_4O_2$: C 38.81, H 4.19, N 25.86; found: C 38.75, H 4.16, N 25.91.

2-Chloro-4-methoxy-6-(N-phenylacetamido)-1,3,5-triazine (IV)

N-Phenylacetamide (2.34 g, 17.3 mmol) was added to a suspension of sodium hydride (692 mg, 17.3 mmol, 60% dispersion in oil) in THF (20 mL) at 0 °C. After stirring for 90 min at 40 °C, the reaction mixture was cooled to 0 °C. The mixture was added dropwise to a THF (20 mL) solution of 2,4-dichloro-6-methoxy-1,3,5-triazine (**6**, 2.83 g, 15.7 mmol), and the mixture was stirred at room temperature for 30 min. The reaction mixture was quenched with

sat. aq. NH_4Cl at 0 °C, extracted with AcOEt. The organic layer was washed with brine, dried over $MgSO_4$, filtered, and concentrated under reduced pressure. The residue was purified by column chromatography (hexane/EtOAc 6:1) to afford 2-chloro-4-methoxy-6-(*N*-phenylacetamido)-1,3,5-triazine (**IV**) (2.76 g, 63%) as a white powder. M.p. 83–84 °C; ^{1}H NMR (400 MHz, $CDCl_3$, TMS): δ=2.64 (s, 3H), 3.90 (s, 3H), 7.16 (m, 2H), 7.41 (m, 1H), 7.47 (m, 2H); ^{13}C NMR (100 MHz, $CDCl_3$): δ=27.53, 55.99, 128.62, 128.65, 129.64, 139.24, 168.45, 171.34, 171.84, 172.55; IR (KBr): 3014, 2956, 1712, 1558, 1523, 1427, 1360, 1225, 1198, 1178, 1016, 912 cm^{-1}; HRMS (EI-Sector): *m*/*z* $[M]^+$ calcd for $C_{12}H_{11}ClN_4O_2$: 278.0571; found: 278.0576; elemental analysis calcd (%) for $C_{12}H_{11}ClN_4O_2$: C 51.72, H 3.98, N 20.10; found: C 51.46, H 4.02, N 20.03.

2-Chloro-4-methoxy-6-(N-methylbenzamido)-1,3,5-triazine (V)

N-Methylbenzamide (324 mg, 2.40 mmol) was added to a suspension of sodium hydride (88 mg, 2.2 mmol, 60% dispersion in oil) in THF (4 mL) at 0 °C. After stirring for 30 min at 50 °C, the reaction mixture was cooled to 0 °C. 2,4-Dichloro-6-methoxy-1,3,5-triazine (**6**) (360 mg, 2.00 mmol) was added to the suspension and the mixture was stirred for 43 h at room temperature. The reaction mixture was quenched with sat. aq. NH_4Cl at 0 °C, extracted with AcOEt. The organic layer was washed with brine, dried over Na_2SO_4, filtered, and concentrated under reduced pressure. The residue was purified by column chromatography (hexane/AcOEt 6:1) to afford 2-chloro-4-methoxy-6-(*N*-methylbenzamido)-1,3,5-triazine (**V**) (467 mg, 84%) as a white powder. M.p. 74–75 °C; ^{1}H NMR (400 MHz, $CDCl_3$, TMS): δ=3.38 (s, 3H) , 3.61 (s, 3H), 7.37(m, 2H), 7.47 (m, 1H), 7.53 (m, 2H); ^{13}C NMR (100 MHz, $CDCl_3$): δ=34.00, 55.44, 128.07, 128.52, 131.64, 136.84, 168.60, 170.38, 171.65, 173.42; IR (KBr): 3005, 2949, 1695, 1568, 1508, 1448, 1402, 1375, 1363, 1248, 1030, 1016, 806, 731, 700 cm^{-1}; HRMS (EI-sector): *m*/*z* $[M+H]^+$ calcd for $C_{12}H_{11}ClN_4O_2$: 278.0571; found: 278.0570; elemental analysis calcd (%) for $C_{12}H_{11}ClN_4O_2$: C 51.72, H 3.98, N 20.10; found: C 51.77, H 4.05, N 20.15.

2-Chloro-4-methoxy-6-(N-phenylbenzamido)-1,3,5-triazine (VI)

N-Phenylbenzamide (5.00 g, 25.3 mmol) in THF (25 mL) was added to the suspension of sodium hydride (1.22 g, 30.4 mmol, 60% dispersion in oil) in THF (10 mL) at 0 °C. After stirring for 2.5 h at 50 °C, the reaction mixture was cooled to 0 °C. A THF (7 mL) solution of 2,4-dichloro-6-methoxy-1,3,5-triazine (**6**, 5.48 g, 30.4 mmol) was added dropwise to the suspension and the reaction mixture was stirred for 1 h at 0 °C. AcOEt was added to the mixture, and the mixture was washed with sat. aq. NH_4Cl and brine. The organic layer was dried over Na_2SO_4, filtered, and concentrated. The residue was purified by column chromatography (hexane/EtOAc 8:2) to afford 2-chloro-4-methoxy-6-(*N*-phenybenzamido)-1,3,5-triazine (**VI**) (7.07 g, 82%) as a white

powder. M.p. 134–135 °C; ^{1}H NMR (400 MHz, $CDCl_3$, TMS): δ=3.60 (s, 3H), 7.28–7.30 (m, 2H), 7.38–7.54 (m, 6H), 7.74–7.76 (m, 2H); ^{13}C NMR (100 MHz, $CDCl_3$): δ=55.75, 128.08, 128.43, 128.78, 129.08, 129.71, 132.57, 135.61, 139.15, 169.02, 170.99, 171.94, 172.40; IR (KBr): 3099, 3062, 3037, 2997, 2951, 1693, 1597, 1560, 1522, 1512, 1433, 1375, 1294, 1269, 1255, 1234 cm^{-1}; HRMS (EI-sector): *m*/*z* $[M]^+$ calcd for $C_{17}H_{13}ClN_4O_2$: 340.0727; found: 340.0729; elemental analysis calcd (%) for $C_{17}H_{13}ClN_4O_2$: C 59.92, H 3.85, N 16.44; found: C 59.89, H 3.77, N 16.34.

2-Acetamido-4,6-dichloro-1,3,5-triazine (7a)

Acetamide (710 mg, 12.0 mmol) in THF (10 mL) was added to a suspension of sodium hydride (480 mg, 12.0 mmol, 60% dispersion in oil) in THF (20 mL) at 0 °C. After stirring for 3 h at 50 °C, the reaction mixture was cooled to −78 °C. Cyanuric chloride (1.00 g, 5.42 mmol) in THF (7.5 mL) was added to the suspension, and the mixture was stirred for 15 h at −78 °C. AcOH (0.69 mL, 12 mmol) in THF (5 mL) was added at −78 °C, and the suspension was diluted with AcOEt. The organic layer was washed with sat. aq. $NaHCO_3$ and brine, dried over Na_2SO_4, filtered, and concentrated under reduced pressure. The residue was purified by column chromatography (hexane/EtOAc 7:3) and recrystallized from CH_2Cl_2/hexane to afford 2-acetamido-4,6-dichloro-1,3,5-triazine (**7a**, 411 mg, 36%) as a white powder. M.p. 187–188 °C; ^{1}H NMR (400 MHz, $CDCl_3$, TMS) δ=2.61 (s, 3H), 8.38 (br s, 1H); ^{13}C NMR (100 MHz, $CDCl_3$) δ=26.05, 164.07, 170.72, 172.05; IR (KBr): 3217, 3099, 3030, 1709, 1579, 1539, 1475, 1406, 1290, 1234, 845, 804 cm^{-1}; HRMS (DART-TOF): *m*/*z* $[M+H]^+$ calcd for $C_5H_5Cl_2N_4O_1$: 206.9840; found: 206.9865; elemental analysis calcd (%) for $C_5H_4Cl_2N_4O$: C 29.01, H 1.95, N 27.06; found: C 29.18, H 2.20, N 27.22.

2-Benzamido-4,6-dichloro-1,3,5-triazine (7b)

Benzamide (1.45 g, 12.0 mmol) in THF (10 mL) was added to a suspension of sodium hydride (480 mg, 12.0 mmol, 60% dispersion in oil) in THF (20 mL) at 0 °C. After stirring for 50 min at 50 °C, the reaction mixture was cooled to −78 °C. Cyanuric chloride (1.00 g, 5.47 mmol) in THF (5 mL) was added to the suspension, and the mixture was stirred for 5 h at −78 °C. AcOH (0.69 mL, 12 mmol) in THF (5 mL) was added at −78 °C, and the suspension was diluted with AcOEt. The organic layer was washed with sat. aq. $NaHCO_3$ and brine, dried over Na_2SO_4, filtered, and concentrated under reduced pressure. The residue was purified by column chromatography (hexane/EtOAc 7:3) to afford 2-benzamido-4,6-dichloro-1,3,5-triazine (**7b**, 955 mg, 65%) as a white powder. M.p. 184–185 °C; ^{1}H NMR (400 MHz, $CDCl_3$, TMS) δ=7.52–7.56 (m, 2H), 7.63–7.68 (m, 1H), 7.96–7.98 (m, 2H), 9.04 (br s, 1H); ^{13}C NMR (100 MHz, $CDCl_3$) δ=128.08, 129.31, 132.44, 133.91, 164.07, 164.73, 172.29; IR (KBr): 3217, 3132, 3103, 1709, 1576, 1535, 1473, 1408, 1298, 1232, 1111, 1099, 843, 804 cm^{-1}; HRMS (DART-TOF): *m*/*z* $[M+H]^+$ calcd (%) for

$C_{10}H_7Cl_2N_4O_1$: 268.9997; found: 269.0019; elemental analysis calcd (%) for $C_{10}H_6Cl_2N_4O$: C 44.64, H 2.25, N 20.82; found: C 44.54, H 2.25, N 20.91.

2-Acetamido-4-chloro-6-methoxy-1,3,5-triazine (I)

2-Acetamido-4,6-dichloro-1,3,5-triazine (**7**a, 50 mg, 0.24 mmol) was added to a solution of 1,10-phenanthroline (87 mg, 0.49 mmol) in MeOH (1.2 mL) at room temperature. After stirring for five days, the solution was concentrated under reduced pressure. The residue was purified by column chromatography (hexane/EtOAc 7:3) to afford 2-acetamido-4-chloro-6-methoxy-1,3,5-triazine (**I**, 33 mg, 67%) as a white powder. M.p. 149–150 °C; ^{1}H NMR (400 MHz, $CDCl_3$, TMS) δ=2.60 (s, 3H), 4.07 (s, 3H), 8.10 (br s, 1H); ^{13}C NMR (100 MHz, $CDCl_3$) δ=26.02, 56.29, 164.89, 171.08, 171.75, 172.34; IR (KBr): 3221, 3126, 1712, 1684, 1577, 1485, 1362, 1290, 1252, 1038, 810 cm^{-1}; HRMS (DART-TOF): *m*/*z* $[M+H]^+$ calcd for $C_6H_8Cl_1N_4O_2$: 203.0336; found: 203.0337.

2-Benzamido-4-chloro-6-methoxy-1,3,5-triazine (II)

N-(4,6-Dichloro-1,3,5-triazin-2-yl)benzamide (**7b**, 50 mg, 0.19 mmol) was added to a solution of 1,10-phenanthroline (68 mg, 0.38 mmol) in MeOH (935 μL, 0.2 M) at 0 °C. After refluxing for 24 h, the solution was concentrated under reduced pressure. The residue was purified by column chromatography (hexane/EtOAc 7:3) to afford 2-benzamido-4-chloro-6-methoxy-1,3,5-triazine (**II**, 39 mg, 78%) as a white powder. M.p. 118–119 °C; ^{1}H NMR (400 MHz, $CDCl_3$/TMS) δ=4.10 (s, 3H), 7.51–7.55 (m, 2H), 7.61–7.65 (m, 1H), 7.89–7.91 (m, 2H), 8.60 (br s, 1H); ^{13}C NMR (100 MHz, $CDCl_3$) δ=56.31, 127.85, 129.18, 133.13, 133.44, 164.39, 165.41, 172.11, 172.44; IR (KBr): 3259, 2951, 1716, 1697, 1581, 1541, 1481, 1406, 1362, 1286, 1259, 1207, 1128, 812, 706 cm^{-1}; HRMS (DART-TOF): *m*/*z* $[M+H]^+$ calcd for $C_{11}H_{10}Cl_1N_4O_2$: 265.0492; found: 265.0468. Crystals suitable for single-crystal X-ray structure analysis were obtained from THF–hexane, 4:1.

4-Methyl-4-(4-methoxy-6-(N-methylacetamido)-1,3,5-triazin-2-yl)morpholinium perchlorate (VII)

NMM (335 μL, 3.05 mmol) was added to a THF (9 mL) solution of 2-chloro-4-methoxy-6-(*N*-methylacetamido)-1,3,5-triazine (**III**, 600 mg, 2.77 mmol) and lithium perchlorate (324 mg, 3.05 mmol) at room temperature. After stirring for 2 h, the mixture was cooled to 0 °C. The resulting precipitate was collected by suction, washed with THF, and dried under reduced pressure to give 4-methyl-4-(4-methoxy-6-(*N*-methylacetamido)-1,3,5-triazin-2-yl)morpholinium perchlorate (**VII**, 1.05 g, 98%) as a white powder. M.p. 182–183 °C; ^{1}H NMR (400 MHz, DMSO-d_6, TMS): δ=2.63 (s, 3H), 3.39 (s, 3H), 3.47 (s, 3H), 3.78 (m, 2H), 3.89 (m, 2H), 4.01 (m, 2H), 4.10 (s, 3H), 4.35 (m, 2H); ^{13}C NMR (100 MHz, DMSO-d_6): δ=27.66, 32.43, 55.18, 56.70, 59.51,

61.39, 168.48, 168.96, 171.80, 172.72; IR (KBr): 3045, 3025, 2992, 2964, 2889, 2875, 1707, 1610, 1541, 1518, 1475, 1444, 1379, 1369, 1288, 1122, 1095, 1011 cm^{-1}; HRMS (FAB-sector): *m*/*z* $[M–ClO_4^-]^+$ calcd for $C_{12}H_{20}N_5O_3$: 282.1561; found: 282.1565.

4-Methyl-4-(4-methoxy-6-(N-phenylacetamido)-1,3,5-triazin-2-yl)morpholinium perchlorate (VIII)

NMM (126 µL, 1.14 mmol) was added to a THF (3.5 mL) solution of 2-chloro-4-methoxy-6-(*N*-phenybenzamido)-1,3,5-triazine (**IV**, 290 mg, 1.04 mmol) and lithium perchlorate (121 mg, 1.14 mmol) at room temperature. After stirring for 45 min, Et_2O was added to the mixture at 0 °C. The resulting precipitate was collected by suction, washed with Et_2O, and dried under reduced pressure to give 4-methyl-4-(4-methoxy-6-(*N*-phenylacetamido)-1,3,5-triazin-2-yl)morpholinium perchlorate (**VIII**, 419 mg, 91%) as a white powder. M.p. 149–150 °C; ^1H NMR (400 MHz, DMSO-d_6, TMS): δ=2.71 (s, 3H), 3.42 (s, 3H), 3.69 (m, 2H), 3.81–3.94 (m, 4H), 3.97 (s, 3H), 4.13 (m, 2H), 7.27 (m, 2H), 7.42(m, 1H), 7.49 (m, 2H); ^{13}C NMR (100 MHz, DMSO-d_6): δ=27.50, 54.41, 56.58, 59.48, 61.28, 128.22, 128.53, 129.22, 139.21, 168.66, 168.84, 171.93, 172.66; IR (KBr): 3055, 2960, 2887, 1697, 1626, 1541, 1508, 1435, 1385, 1365, 1261, 1093 cm^{-1}; HRMS (EI-sector): *m*/*z* $[M–ClO_4^-]^+$ calcd for $C_{17}H_{22}N_5O_3$: 344.1717; found: 344.1716. Crystals suitable for single-crystal X-ray structure analysis were obtained from CH_3CN–hexane, 4:1.

4-Methyl-4-(4-methoxy-6-(N-methylbenzamido)-1,3,5-triazin-2-yl)morpholinium chloride (IX)

NMM (495 µL, 4.50 mmol) was added to a solution of 2-chloro-4-methoxy-6-(*N*-methylbenzamido)-1,3,5-triazine (**V**, 500 mg, 1.79 mmol) in Et_2O (9 mL) at 0 °C. After stirring for 3 h, the resulting precipitate was collected by suction, washed with Et_2O, and dried under reduced pressure to give 4-methyl-4-(4-methoxy-6-(*N*-methylbenzamide)-1,3,5-triazine-2-yl)morpholinium chloride monohydrate (**IX**, 642 mg, 90%, water content 4.5% w/w) as a white powder. M.p. 99–100 °C; ^1H NMR (400 MHz, CD_3OD, TMS): δ=3.22 (s, 3H), 3.57 (m, 4H), 3.66 (s, 3H), 3.82 (m, 2H), 3.90 (m, 2H), 3.96 (s, 3H), 7.49 (m, 2H), 7.58 (m, 1H), 7.67 (m, 2H); ^{13}C NMR (100 MHz, CD_3OD): δ=34.54, 56.34, 57.25, 60.94, 63.12, 129.35, 130.05, 133.24, 138.09, 169.83, 170.50, 173.75, 174.38; IR (KBr): 3542, 3379, 3340, 3275, 3028, 2954, 1685, 1658, 1604, 1545, 1522, 1471, 1387, 1313, 1290, 1223, 1111 cm^{-1}; HRMS (FAB-sector): *m*/*z* $[M–Cl^-–H_2O]^+$ calcd for $C_{17}H_{22}ClN_5O_3$: 344.1717; found: 344.1724; elemental analysis calcd (%) for $C_{17}H_{24}ClN_5O_4$ (monohydrate): C 51.32, H 6.08, N 17.60; found: C 50.94, H 6.03, N 17.53.

4-Methyl-4-(4-methoxy-6-(N-phenylbenzamido)-1,3,5-triazin-2-yl)morpholinium chloride (X)

NMM (484 μL, 4.40 mmol) was added to a Et_2O (20 mL), THF (4 mL), and H_2O (146 μL, 8.10 mmol) solution of 2-chloro-4-methoxy-6-(*N*-phenybenzamido)-1,3,5-triazine (**VI**, 1.00 g, 2.93 mmol) at 0 °C. After stirring for 3.5 h at room temperature, Et_2O (20 mL) was added. The resulting precipitate was collected by suction, washed with Et_2O, and dried under reduced pressure to give 4-methyl-4-(4-methoxy-6-(*N*-phenylbenzamido)-1,3,5-triazin-2-yl)morpholinium chloride dihydrate (**X**, 1.12 g, 80%, water content 3.8% w/w) as a white powder. M.p. 91–92 °C; ^{1}H NMR (400 MHz, CD_3OD, TMS) δ=3.30 (s, 3H), 3.53–3.69 (m, 4H), 3.77 (s, 3H), 3.83 (m, 2H), 4.00 (m, 2H), 7.43–7.65 (m, 8H), 7.89–7.90 (m, 2H); ^{13}C NMR (100 MHz, CD_3OD): δ=56.51 57.15, 61.11, 63.06, 129.33, 129.78, 130.25, 130.27, 130.66, 134.19, 136.64, 140.18, 170.29, 170.65, 173.29, 173.84; IR ($CHCl_3$): 3425, 3037, 2916, 2860, 1716, 1610, 1593, 1522, 1437, 1383, 1238, 1144 cm^{-1}; HRMS (FAB-sector): *m*/*z* $[M–Cl^-–2H_2O]^+$ calcd for $C_{22}H_{24}N_5O_3$: 406.1874; found: 406.1871; elemental analysis calcd (%) for $C_{22}H_{28}ClN_5O_5$ (dihydrate): C 55.29, H 5.91, N 14.65; found: C 55.10, H 5.78, N 14.58. Crystals suitable for single-crystal X-ray structure analysis were obtained from CH_3CN–MeOH–Et_2O, 3:1:1.

General procedure for condensation of carboxylic acids and amines

***N-Phenethyl-3-phenylpropanamide (3a)*[1,2]: X** (194 mg, 0.44 mmol) was added to a mixture of 3-phenylpropinoic acid **1a** (60 mg, 0.40 mmol) and 2-phenylethylamine **2a** (55 μL, 0.44 mmol) in THF (2.0 mL). After stirring for 3 h at room temperature, the reaction mixture was quenched with 1 M $KHSO_4$, and was concentrated under reduced pressure to remove THF. The mixture was diluted with AcOEt, and washed with 0.5 M HCl, sat. aq. $NaHCO_3$, and brine. The organic layer was dried over Na_2SO_4, filtered, and concentrated under reduced pressure. The residue was purified by column chromatography (hexane/AcOEt 7:3) to afford *N*-phenethyl-3-phenylpropanamide (**3a**, 90 mg, 89%) as a white powder. The yield in MeOH was 95%. M.p. 97–98 °C (lit.[2] m.p. 96–97.5 °C) ; ^{1}H NMR (400 MHz, $CDCl_3$, TMS): δ=2.42 (t, *J*=7.8 Hz, 2H), 2.73 (t, *J*=6.9 Hz, 2H), 2.94 (t, *J*=7.8 Hz, 2H), 3.47 (dt, *J*=6.9, 6.9 Hz, 2H), 5.36 (br s, 1H), 7.07–7.30 (m, 10H); ^{13}C NMR (100 MHz, $CDCl_3$): δ=31.81, 35.77, 38.67, 40.67, 126.38, 126.61, 128.50, 128.66, 128.74, 128.87, 138.98, 140.98, 172.12; HRMS (DART–TOF): m/z $[M+H]^+$ calcd for $C_{17}H_{20}N_1O_1$: 254.1545; found: 254.1516.

***N-Phenyl-3-phenylpropanamide (3b)*[3,4]**

Silica gel column chromatography; hexane/AcOEt 85:15. The yields in MeOH and THF were 80% and 75%, respectively. A white powder; m.p. 96–97 °C (lit.[3] m.p. 98 °C); ^{1}H NMR (600 MHz, $CDCl_3$, TMS): δ=2.66 (t, *J*=7.6 Hz, 2H), 3.06 (t, *J* = 7.6 Hz, 2H), 7.02 (br s, 1H) 7.09 (m, 1H), 7.22–7.25 (m, 3H), 7.28–7.32 (m, 4H), 7.43 (m, 2H); ^{13}C NMR (100 MHz, $CDCl_3$):

δ=31.69, 39.59, 120.07, 124.44, 126.52, 128.52, 128.78, 129.09, 137.84, 140.75, 170.54; HRMS (DART–TOF): *m*/*z* $[M+H]^+$ calcd for $C_{15}H_{16}N_1O_1$: 226.1232; found: 226.1232.

***N,N*-Diethyl-3-phenylpropanamide (3c)**[1,5]

Silica gel column chromatography; hexane/AcOEt 8:2. The yields in MeOH and THF were 65% and 85%, respectively. A colorless oil; ^{1}H NMR (400 MHz, $CDCl_3$, TMS): δ=1.10 (t, *J*=7.3 Hz, 3H), 1.11 (t, *J*=7.3 Hz, 3H), 2.59 (t, *J*=7.3 Hz, 2H), 2.99 (t, *J*=7.3 Hz, 2H), 3.22 (q, *J*=7.3 Hz, 2H), 3.38 (q, *J*=7.3 Hz, 2H), 7.20–7.31 (m, 5H); ^{13}C NMR (100 MHz, $CDCl_3$): δ=13.21, 14.39, 31.79, 35.24, 40.34, 42.02, 126.20, 128.59, 128.59, 141.70, 171.43; HRMS (DART–TOF): *m*/*z* $[M+H]^+$ calcd for $C_{13}H_{20}N_1O_1$: 206.1545; found: 206.1565.

***N*-Phenethyl-2,2-dimethylpropanamide (3d)**[1,6]

Silica gel column chromatography; hexane/AcOEt 9:1 to 7:3. The yields in MeOH and THF were 87% and 90%, respectively. A white powder; m.p. 81–82 °C (lit.[1] m.p. 81–82 °C); ^{1}H NMR (400 MHz, $CDCl_3$, TMS): δ=1.14 (s, 9H), 2.81 (t, *J*=6.9 Hz, 2H), 3.50 (dt, *J*=6.9, 7.8 Hz, 2H), 5.64 (br s, 1H), 7.18–7.33 (m, 5H); ^{13}C NMR ($CDCl_3$): δ=27.66, 35.79, 38.77, 40.75, 126.62, 128.73, 128.95, 139.18, 178.49; HRMS (DART–TOF): *m*/*z* $[M+H]^+$ calcd for $C_{13}H_{20}N_1O_1$: 206.1545; found: 206.1564.

***N*-Phenethylbenzamide (3e)**[1,6]

Silica gel column chromatography; hexane/AcOEt 70:30. The yields in MeOH and THF were 97% and 95%, respectively. A white powder; m.p. 111–112 °C (lit.[6] 113–116 °C); ^{1}H NMR (400 MHz, $CDCl_3$, TMS): δ=2.95 (t, *J*=6.8 Hz, 2H), 3.73 (dt, *J*=6.8, 6.8 Hz, 2H), 6.10 (br s, 1H), 7.24–7.26 (m, 3H), 7.32–7.35 (m, 2H), 7.39–7.43 (m, 2H), 7.47–7.51 (m, 1H), 7.67-7.70 (m, 2H); ^{13}C NMR ($CDCl_3$): δ=35.84, 41.26, 126.74, 126.93, 128.69, 128.86, 128.96, 131.54, 134.79, 139.04, 167.60; HRMS (DART–TOF): *m*/*z* $[M+H]^+$ calcd for $C_{15}H_{16}N_1O_1$: 226.1232; found: 226.1231.

***N*-Phenethyl-4-nitrobenzamide (3f)**[1,7]

Silica gel column chromatography; hexane/AcOEt 80:20 to 70:30. The yields in MeOH and THF were 91% and 96%, respectively. A white powder; m.p. 149–150 °C (lit.[1] 149.5–150.5 °C); ^{1}H NMR (400 MHz, $CDCl_3$/TMS): δ=2.96 (t, *J*=6.9 Hz, 2H), 3.75 (dt, *J*=6.9, 6.9 Hz, 2H), 6.18 (br s, 1H), 7.23–7.29 (m, 3H), 7.32–7.36 (m, 2H), 7.83 (m, 2H), 8.26 (m, 2H); ^{13}C NMR (100 MHz, $CDCl_3$): δ=35.58, 41.51, 123.94, 126.93, 128.14, 128.88, 128.94, 138.59, 140.31, 149.61, 165.58; HRMS (DART–TOF): *m*/*z* $[M+H]^+$ calcd for $C_{15}H_{15}N_2O_3$: 271.1083; found: 271.1112.

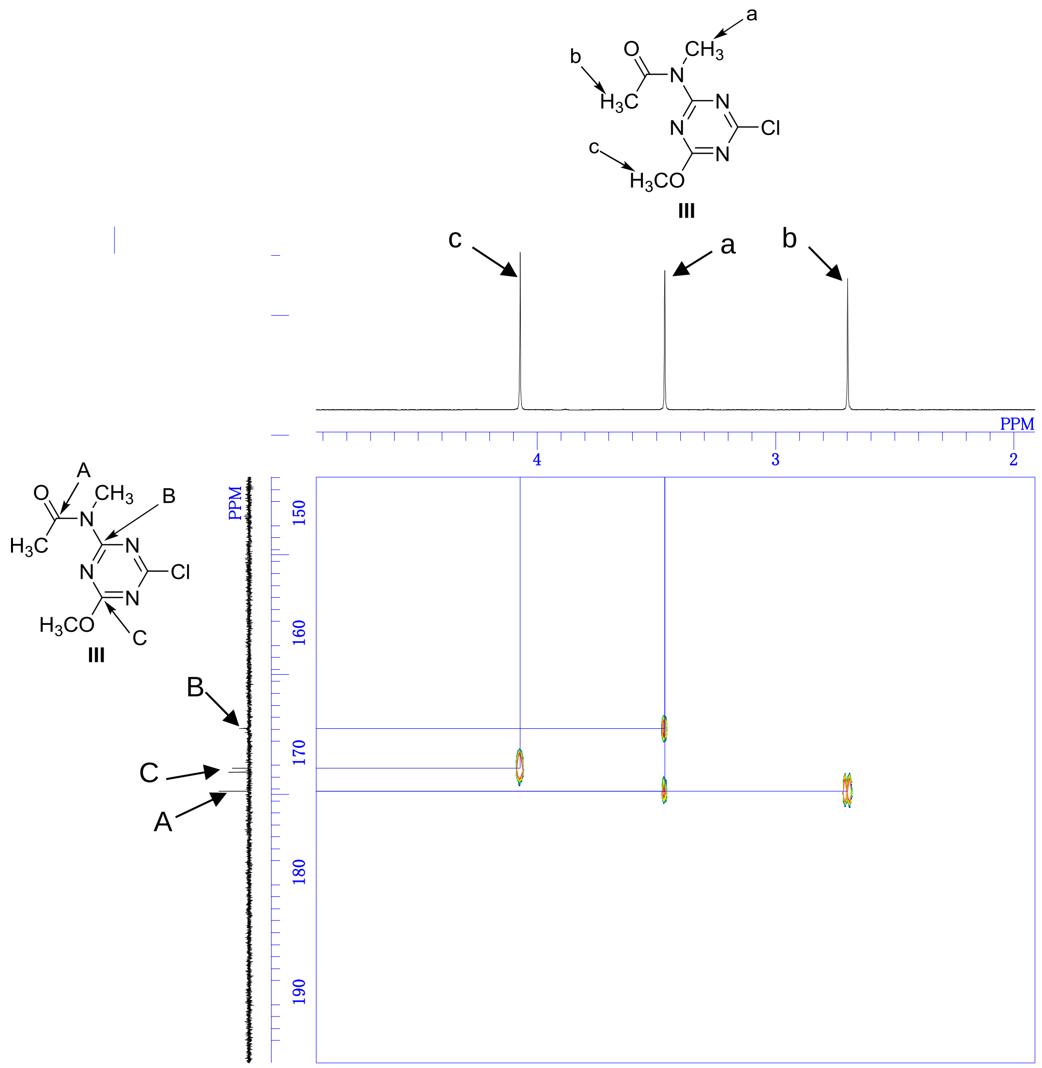

Figure S1: HMBC spectrum of **III**.

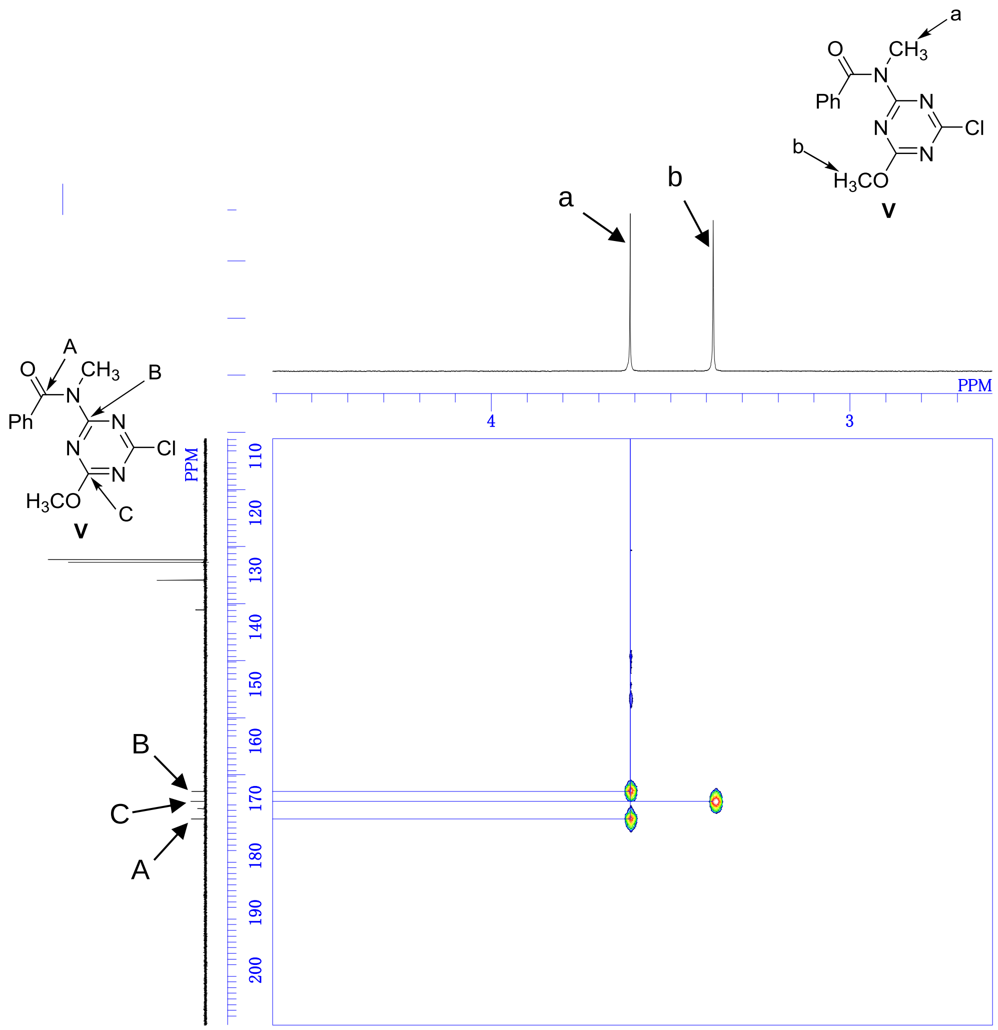

Figure S2: HMBC spectrum of **V**.

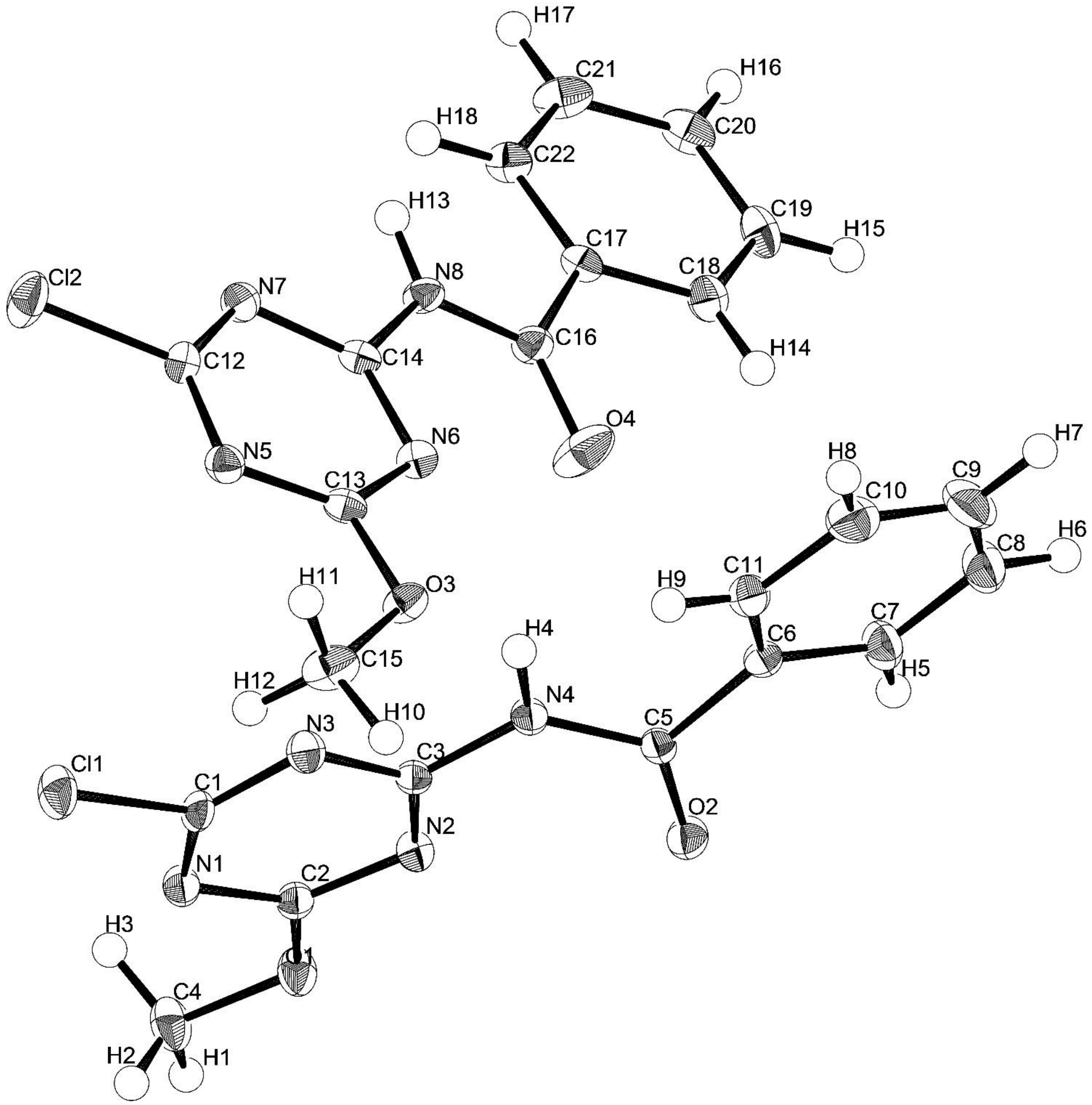

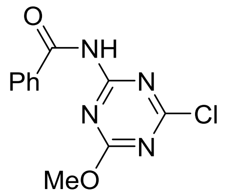

II

Figure S3. Single-crystal X-ray structure analysis of **II** (50% probability ellipsoids).

Crystallographic data of **II** have been deposited with Cambridge Crystallographic Data Centre as supplementary publication no. CCDC 1478426.

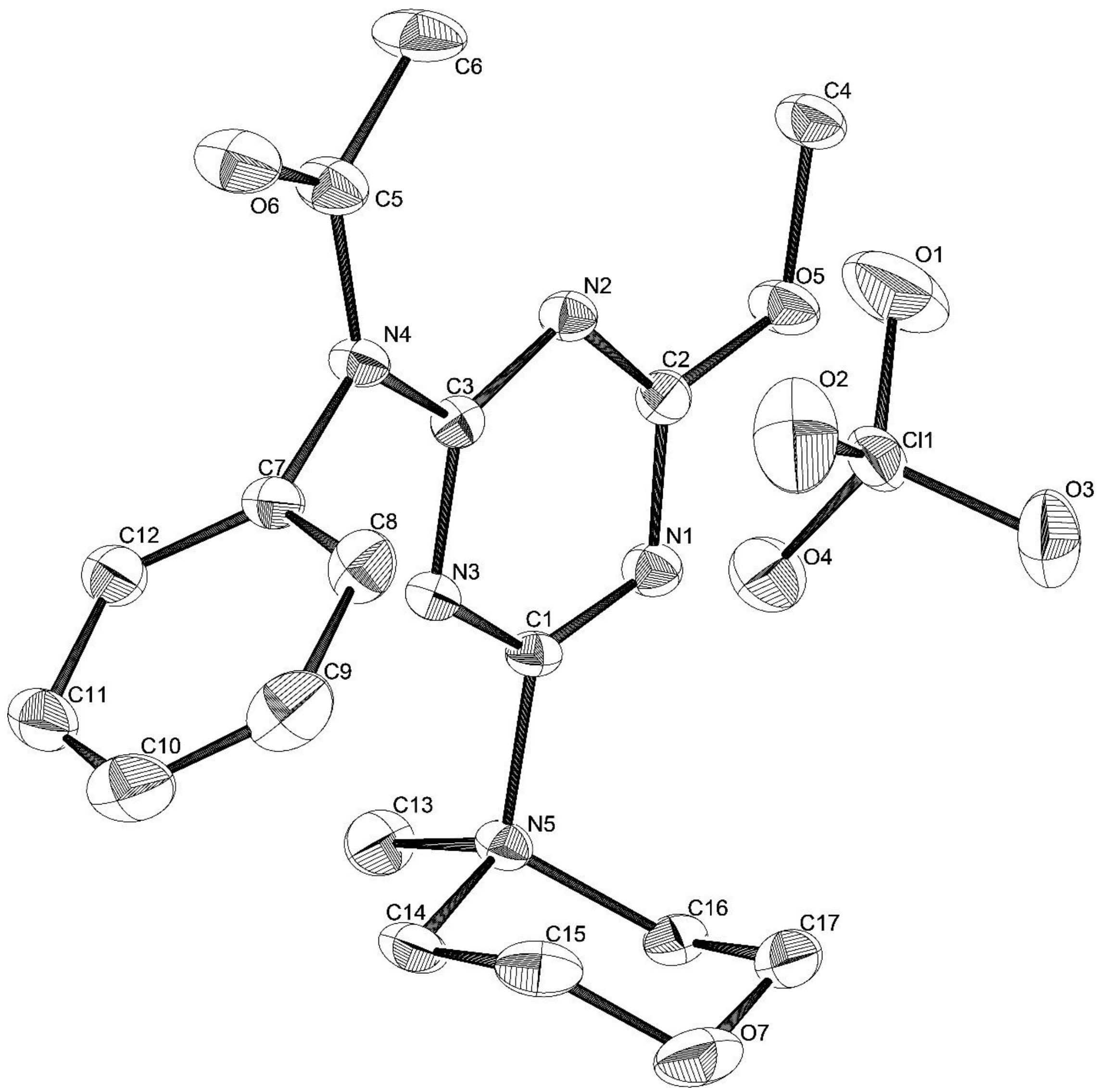

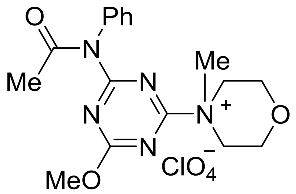

VIII

Figure S4. Single-crystal X-ray structure analysis of **VIII** (50% probability ellipsoids). Hydrogens are omitted for clarity.

Crystallographic data of **VIII** have been deposited with Cambridge Crystallographic Data Centre as supplementary publication no. CCDC 1478427.

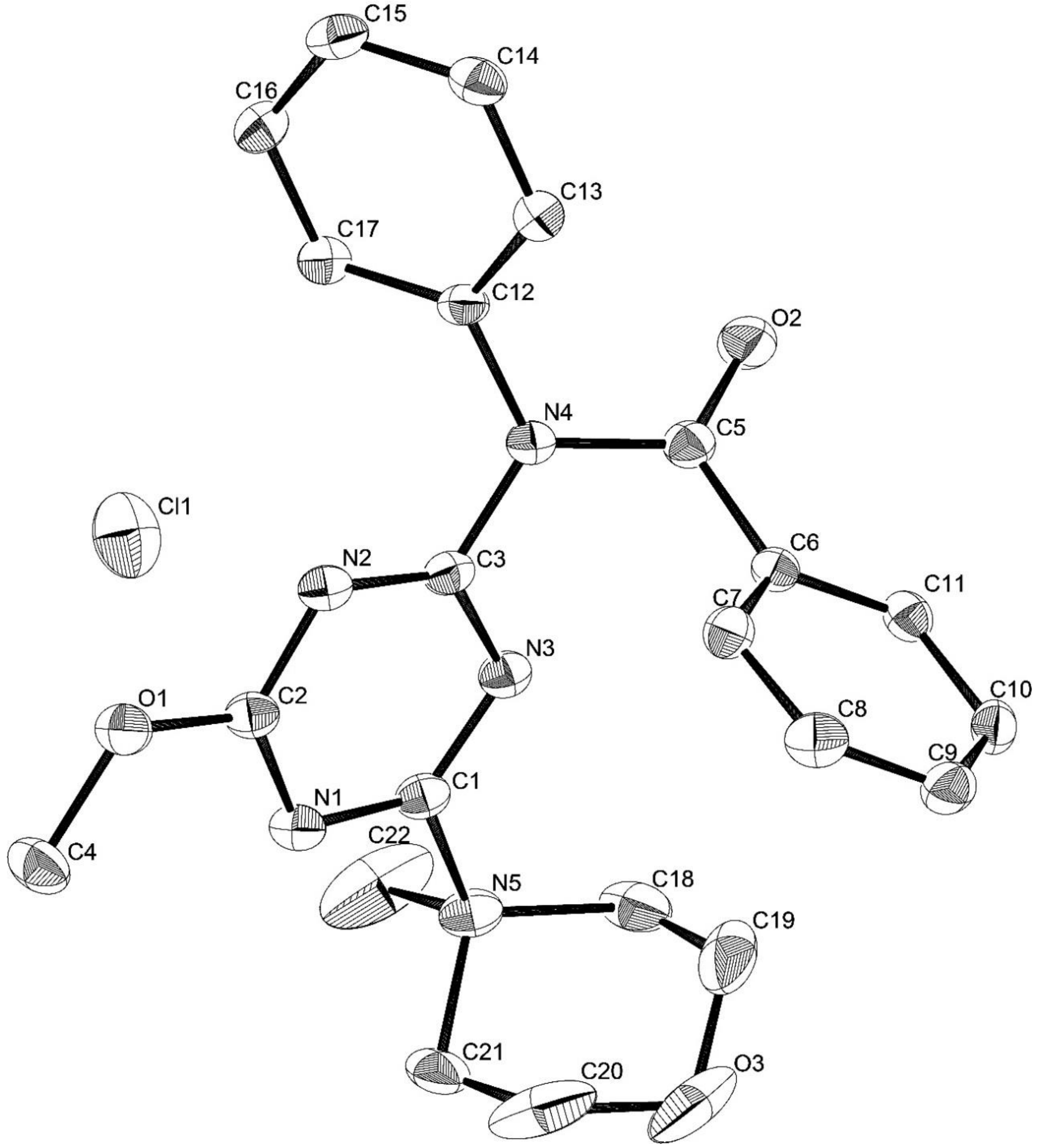

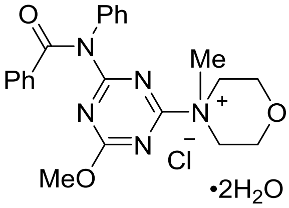

X

Figure S5. Single-crystal X-ray structure analysis of **X** (50% probability ellipsoids). Hydrogens and waters are omitted for clarity.

Crystallographic data of **X** have been deposited with Cambridge Crystallographic Data Centre as supplementary publication no. CCDC 1478428.

Structure determination of 2-acetamido-4-chloro-6-methoxy-1,3,5-triazine (I) by methylation

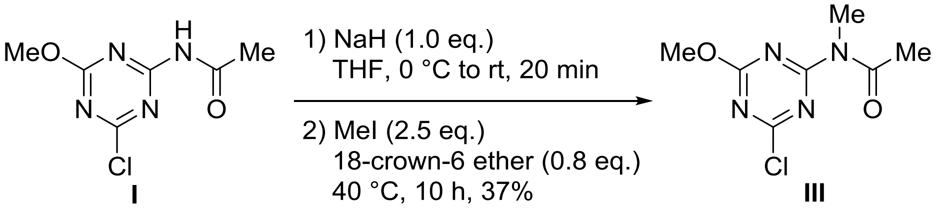

I **III**

2-Acetamido-4-chloro-6-methoxy-1,3,5-triazine (**I**, 30 mg, 0.15 mmol) in THF (360 μL) was added to a suspension of sodium hydride (3.0 mg, 0.14 mmol) in THF (200 μL) at 0 °C. After stirring for 20 min at rt, the reaction mixture was cooled to 0 °C. 18-crown-6 ether (30 mg, 0.11 mmol) and MeI (21 μL, 0.340 mmol) were added to the mixture, and the mixture was stirred for 10 h at 40 °C. To the mixture was added CH_2Cl_2 and 1 M $KHSO_4$. The organic layer was separated, washed with brine (5 mL), dried over Na_2SO_4, filtered, and concentrated under reduced pressure. The residue was purified by column chromatography (hexane/EtOAc 8:2) to afford a white solid (11 mg, 37%) whose ^{1}H NMR, ^{13}C NMR, and HMBC spectra were consistent with 2-chloro-4-methoxy-6-(*N*-methylacetamido)-1,3,5-triazine (**III**). Therefore, structure of **I** is confirmed as indicated in the Scheme.

Attempted synthtsis of I from 2,4-dichloro-6-methoxy-1,3,5-triazine (6)

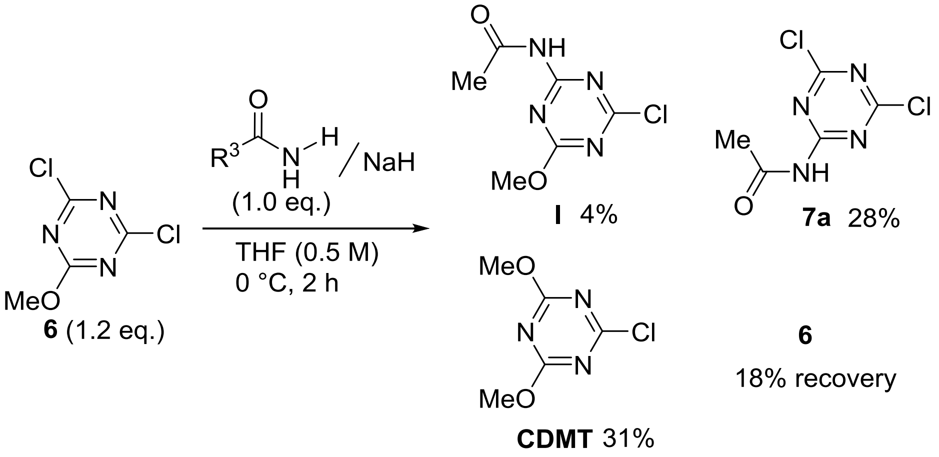

Acetamide (27 mg, 0.46 mmol) in THF (600 µL) was added to a suspension of sodium hydride (20 mg, 0.51 mmol) in THF (300 µL) at 0 °C. After stirring for 45 min at 50 °C, the reaction mixture was cooled to 0 °C. A THF (200 µL) solution of 2,4-dichloro-6-methoxy-1,3,5-triazine (**6**, 100 mg, 0.56 mmol) was added dropwise to the resulting suspension, and the mixture was stirred for 2 h at 0 °C. To the mixture was added CH_2Cl_2 and 1 M $KHSO_4$ at 0 °C. The organic layer was separated, washed with brine, dried over Na_2SO_4, filtered, and concentrated under reduced pressure. The residue was purified by column chromatography (hexane/EtOAc 9:1 to 1:1) to afford **I** (4 mg, 4%), 2-acetamido-4,6-dichloro-1,3,5-triazine (**7a**) (27 mg, 28%), CDMT (25 mg, 31%), and **6** (18 mg, 18% recovery).

Amide-forming reaction with XI in the absence of NMM

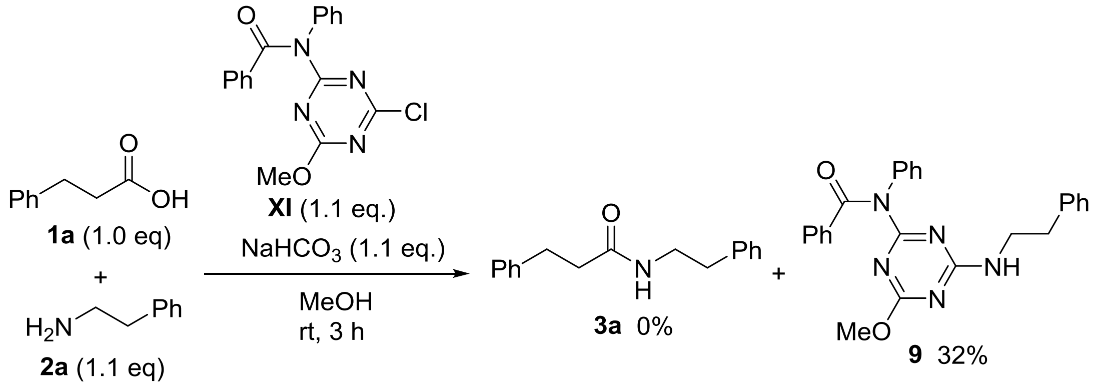

XI (150 mg, 0.44 mmol) was added to a MeOH solution (2.0 mL) of 3-phenylpropinoic acid (60 mg, 0.40 mmol), 2-phenylethylamine (55 μL 0.44 mmol), and $NaHCO_3$ (37 mg, 0.44 mmol) at rt. After stirring for 3 h, 1 M $KHSO_4$ was added and concentrated under reduced pressure to remove MeOH. The suspension was diluted with AcOEt, and the mixture was washed with 0.5 M HCl, sat. aq. $NaHCO_3$ solution, and brine. The organic layer was dried over Na_2SO_4, filtered, and concentrated under reduced pressure. The residue was purified by column chromatography (hexane/AcOEt 7:3) to afford 2-methoxy-4-(*N*-phenethylamino)-6-(*N*-phenybenzamido)-1,3,5-triazine (**9**, 59 mg, 32%) as a white powder. The amide was not observed by ^{1}H NMR.

9: a white powder; m.p. 174–175 °C; ^{1}H NMR (400 MHz, $CDCl_3$, TMS): δ=2.47 (t, *J*=7.0 Hz, 2H major), 2.82 (t, *J*=7.0 Hz, 2H minor), 3.13 (dt, *J*=6.0, 7.0 Hz, 2H major), 3.50 (s, 3H minor), 3.52 (s, 3H major), 3.62 (dt, *J*=6.0, 7.0 Hz, 2H minor), 5.20 (t, *J*=6.0 Hz, 1H minor), 5.36 (t, *J*=6.0 Hz, 1H major), 6.96 (m, 2H major), 7.13 (m, 2H minor), 7.21–7.47 (m, 11H major and 11H minor), 7.67 (m, 2H minor), 7.71 (m, 2H major); ^{13}C NMR (100 MHz, $CDCl_3$): δ 35.54, 35.59, 42.20, 42.23, 54.40, 54.52, 126.55, 126.69, 127.59, 127.66, 128.23, 128.31, 128.38, 128.46, 128.49, 128.62, 128.74, 128.87, 128.89, 129.30, 129.37, 131.31, 137.29, 137.66, 138.63, 138.73, 140.67, 140.73, 166.97, 167.41, 169.01, 169.25, 170.95, 171.28, 173.00, 173.30; IR (KBr): 3370, 3060, 3005, 2949 1695, 1568, 1473, 1448, 1402, 1375, 1363, 1248 cm^{-1}; HRMS (DART–TOF): *m*/*z* [M+H]$^+$ calcd for $C_{25}H_{24}N_5O_2$: 426.1930; found: 426.1918.

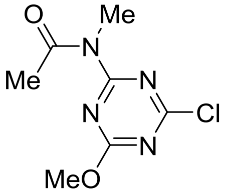
Me
O
N
Me
N
N
Cl
N
MeO

III

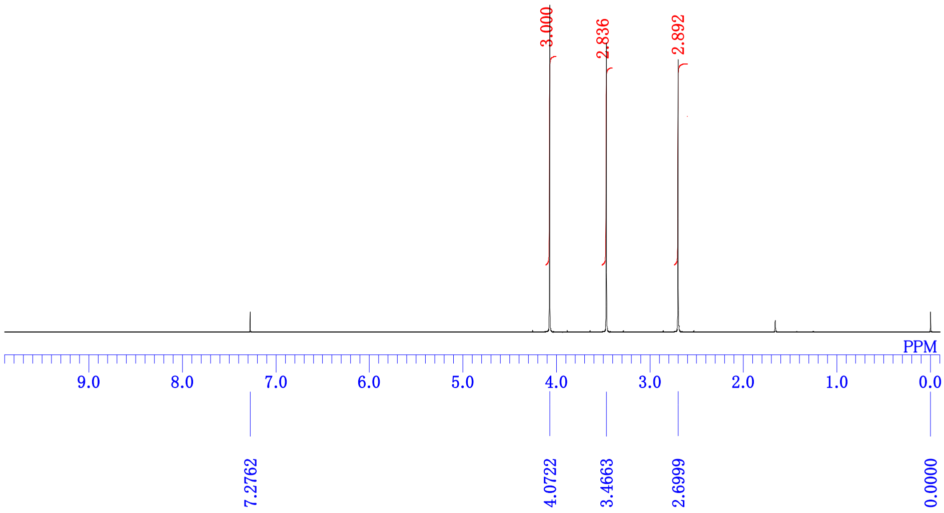
3.000
2.836
2.892
PPM
9.0 8.0 7.0 6.0 5.0 4.0 3.0 2.0 1.0 0.0
7.2762
4.0722
3.4663
2.6999
0.0000

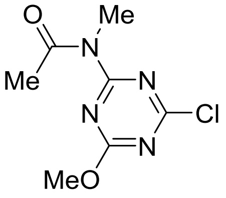
Me
O
N
Me
N
N
Cl
N
MeO

III

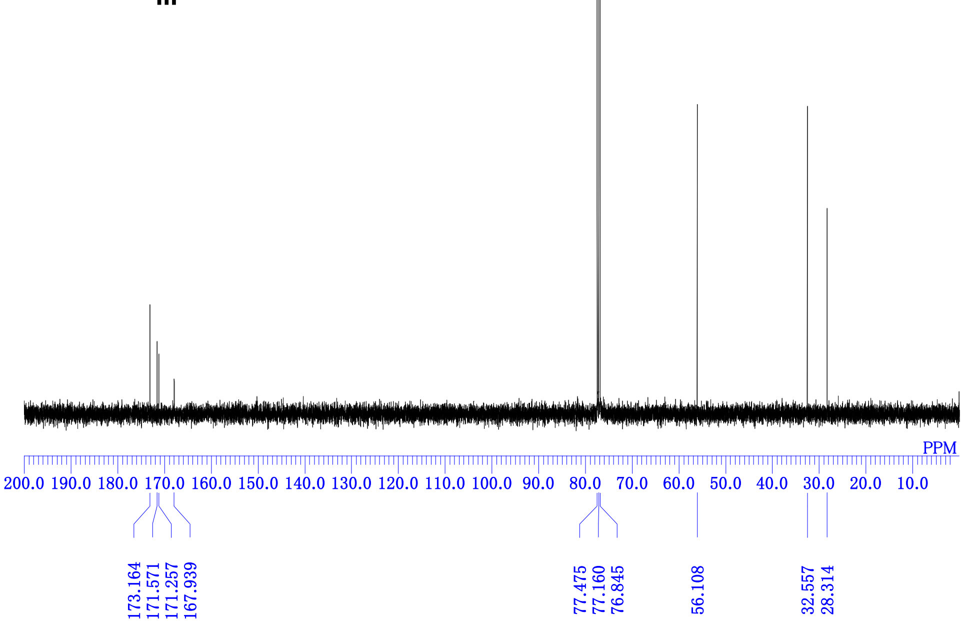
PPM
200.0 190.0 180.0 170.0 160.0 150.0 140.0 130.0 120.0 110.0 100.0 90.0 80.0 70.0 60.0 50.0 40.0 30.0 20.0 10.0
173.164
171.571
171.257
167.939
77.475
77.160
76.845
56.108
32.557
28.314

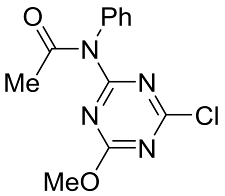
O
Ph
N
Me
N
N
Cl
N
MeO

IV

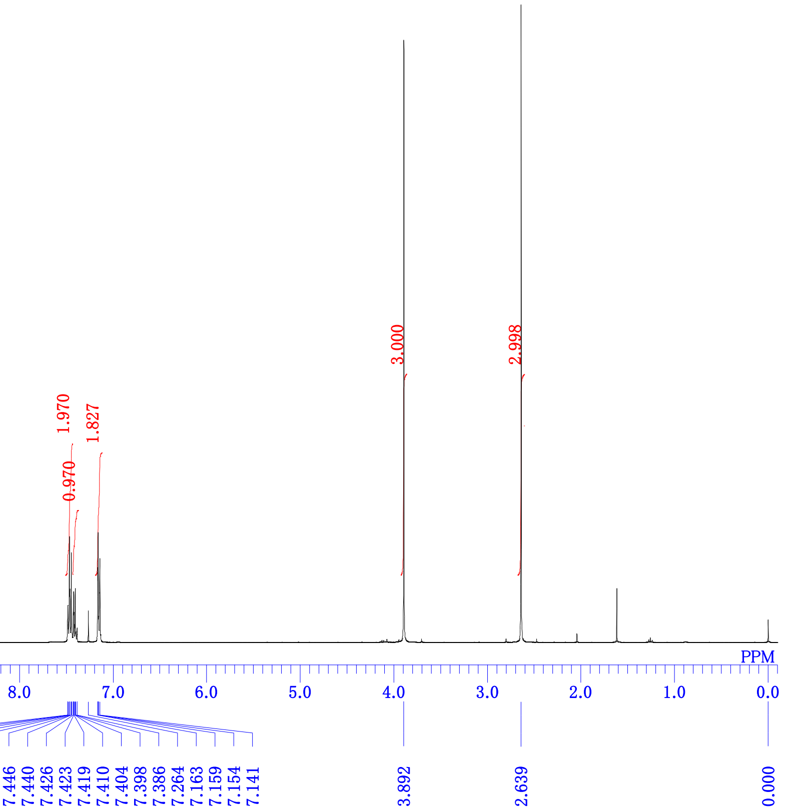
1.970
0.970
1.827
3.000
2.998
PPM
9.0 8.0 7.0 6.0 5.0 4.0 3.0 2.0 1.0 0.0
7.486 7.483 7.478 7.465 7.462 7.446 7.440 7.426 7.423 7.419 7.410 7.404 7.398 7.386 7.264 7.163 7.159 7.154 7.141
3.892
2.639
0.000

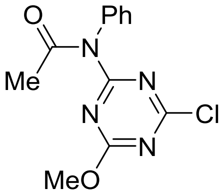
O
Ph
N
Me
N
N
Cl
N
MeO

IV

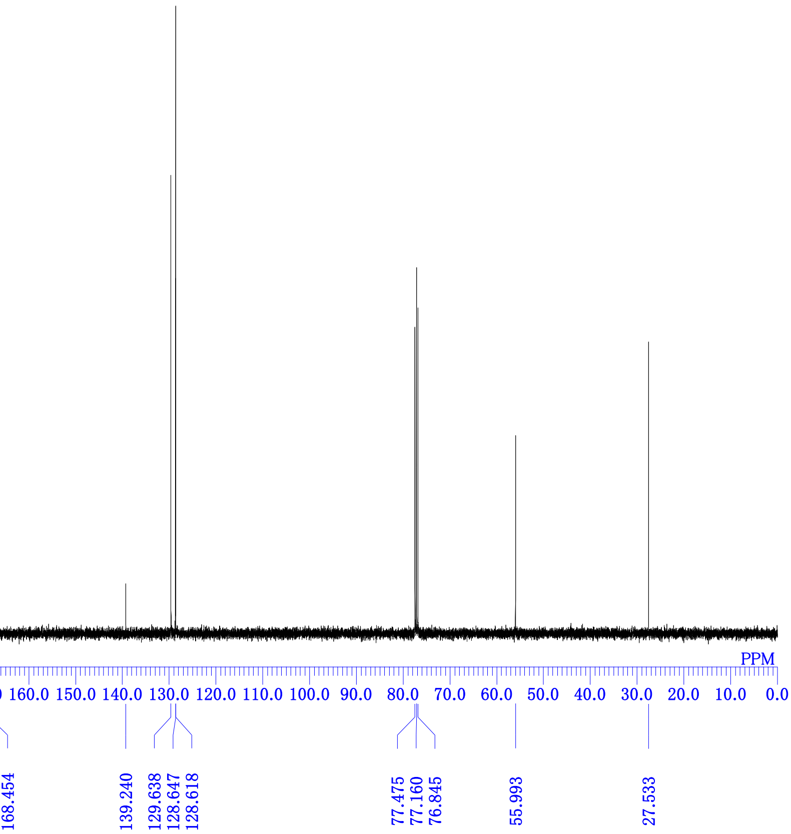
PPM
200.0 190.0 180.0 170.0 160.0 150.0 140.0 130.0 120.0 110.0 100.0 90.0 80.0 70.0 60.0 50.0 40.0 30.0 20.0 10.0 0.0
172.553 171.838 171.343 168.454
139.240
129.638 128.647 128.618
77.475 77.160 76.845
55.993
27.533

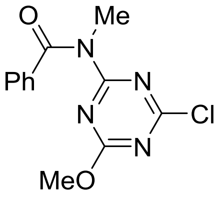

Me
O
N
Ph
N
N
Cl
N
MeO

V

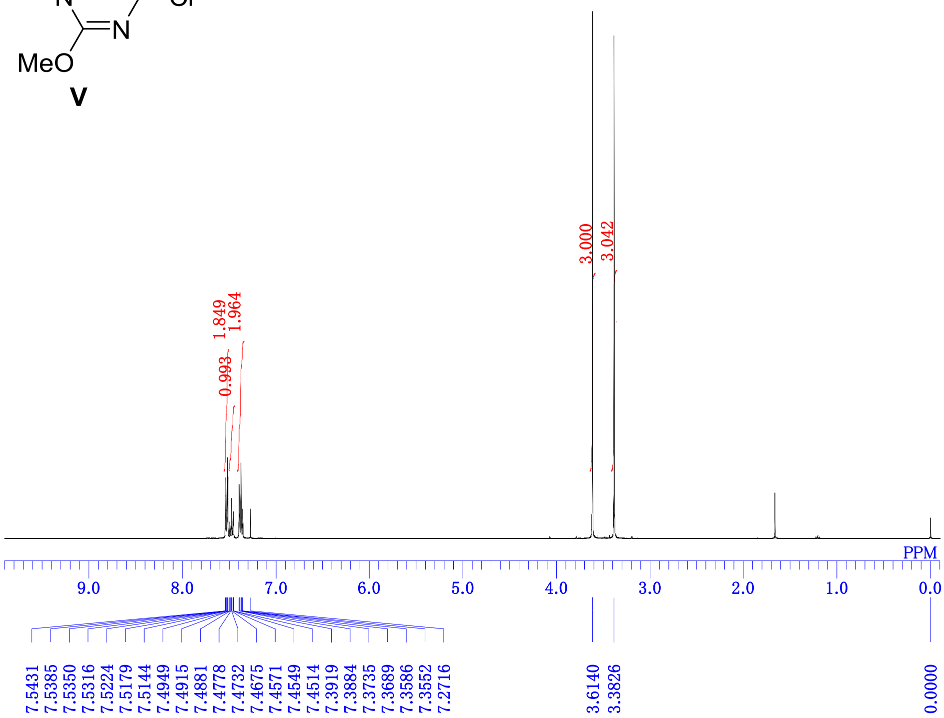

PPM
9.0 8.0 7.0 6.0 5.0 4.0 3.0 2.0 1.0 0.0
0.993 1.849 1.964 3.000 3.042
7.5431 7.5385 7.5350 7.5316 7.5224 7.5179 7.5144 7.4949 7.4915 7.4881 7.4778 7.4732 7.4675 7.4571 7.4549 7.4514 7.3919 7.3884 7.3735 7.3689 7.3586 7.3552 7.2716
3.6140 3.3826
0.0000

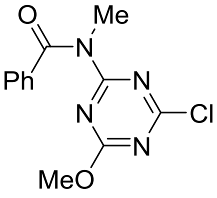

Me
O
N
Ph
N
N
Cl
N
MeO

V

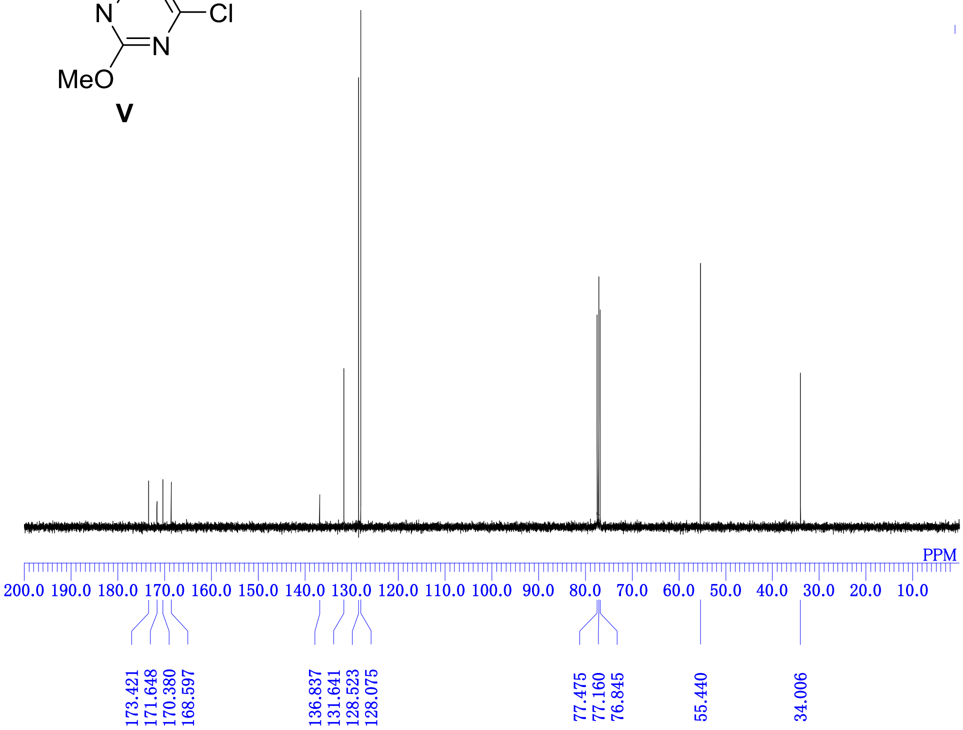

PPM
200.0 190.0 180.0 170.0 160.0 150.0 140.0 130.0 120.0 110.0 100.0 90.0 80.0 70.0 60.0 50.0 40.0 30.0 20.0 10.0
173.421 171.648 170.380 168.597
136.837 131.641 128.523 128.075
77.475 77.160 76.845
55.440
34.006

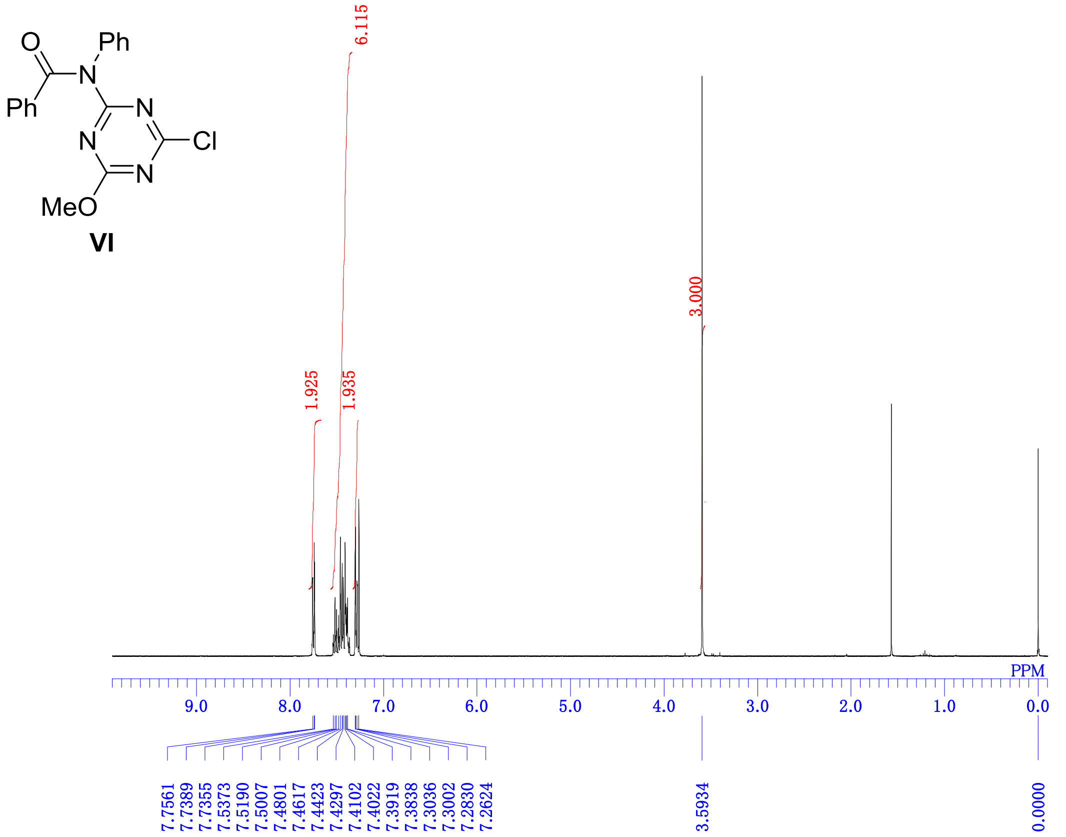
O
Ph
N
Ph
N
N
Cl
N
MeO
1.925
6.115
1.935
3.000
PPM
9.0
8.0
7.0
6.0
5.0
4.0
3.0
2.0
1.0
0.0
7.7561
7.7389
7.7355
7.5373
7.5190
7.5007
7.4801
7.4617
7.4423
7.4297
7.4102
7.4022
7.3919
7.3838
7.3036
7.3002
7.2830
7.2624
3.5934
0.0000

VI

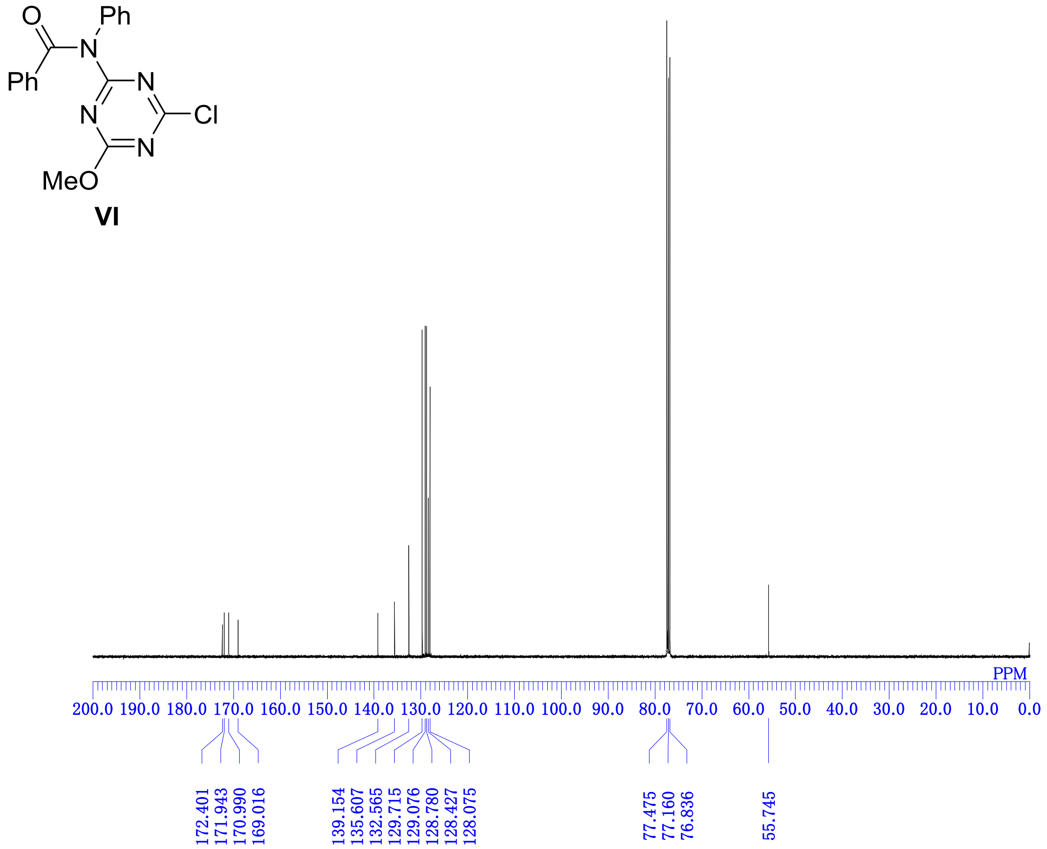
O
Ph
N
Ph
N
N
Cl
N
MeO
PPM
200.0 190.0 180.0 170.0 160.0 150.0 140.0 130.0 120.0 110.0 100.0 90.0 80.0 70.0 60.0 50.0 40.0 30.0 20.0 10.0 0.0
172.401
171.943
170.990
169.016
139.154
135.607
132.565
129.715
129.076
128.780
128.427
128.075
77.475
77.160
76.836
55.745

VI

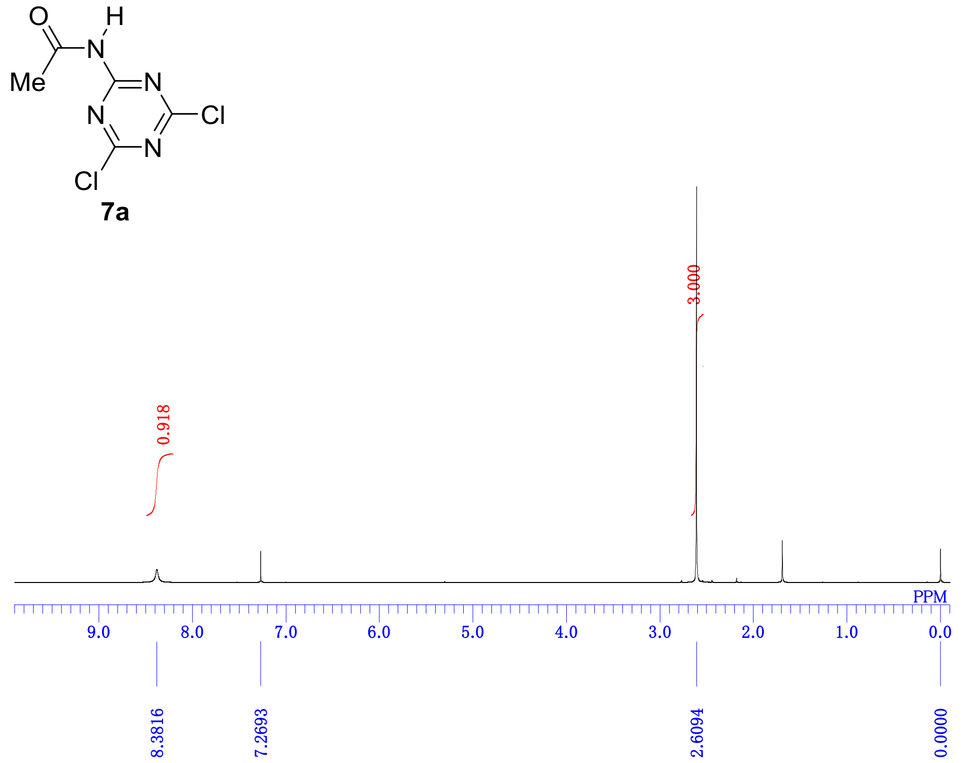
O
H
N
Me
N
N
Cl
N
Cl
7a
0.918
3.000
PPM
9.0
8.0
7.0
6.0
5.0
4.0
3.0
2.0
1.0
0.0
8.3816
7.2693
2.6094
0.0000

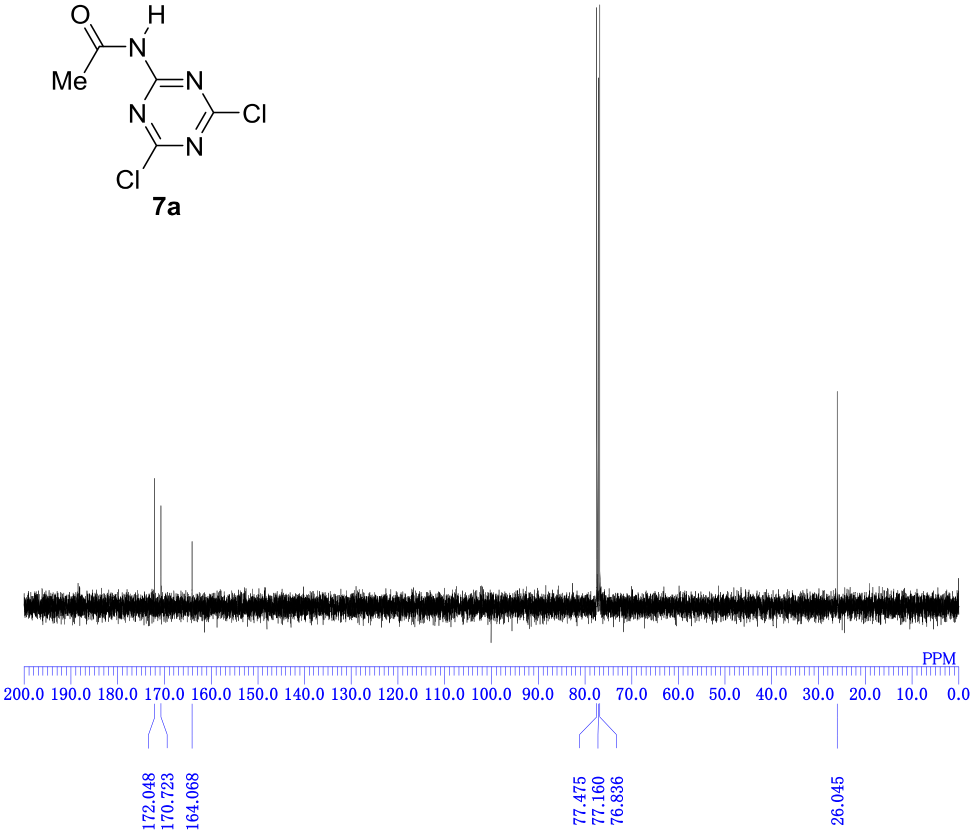
O
H
N
Me
N
N
Cl
N
Cl
7a
PPM
200.0 190.0 180.0 170.0 160.0 150.0 140.0 130.0 120.0 110.0 100.0 90.0 80.0 70.0 60.0 50.0 40.0 30.0 20.0 10.0 0.0
172.048
170.723
164.068
77.475
77.160
76.836
26.045

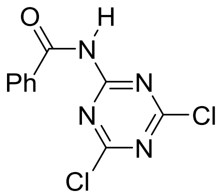
O
H
N
Ph
N
N
Cl
N
Cl

7b

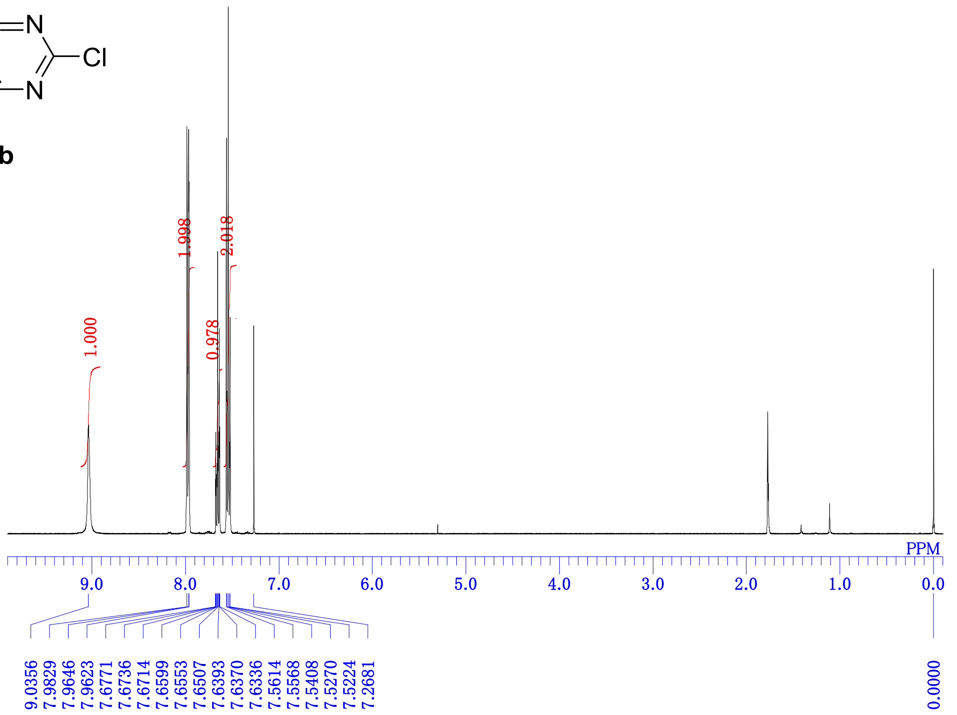
1.000
1.998
0.978
2.018
PPM
9.0 8.0 7.0 6.0 5.0 4.0 3.0 2.0 1.0 0.0
9.0356
7.9829
7.9646
7.9623
7.6771
7.6736
7.6714
7.6599
7.6553
7.6507
7.6393
7.6370
7.6336
7.5614
7.5568
7.5408
7.5270
7.5224
7.2681
0.0000

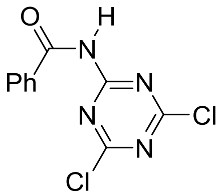
O
H
N
Ph
N
N
Cl
N
Cl

7b

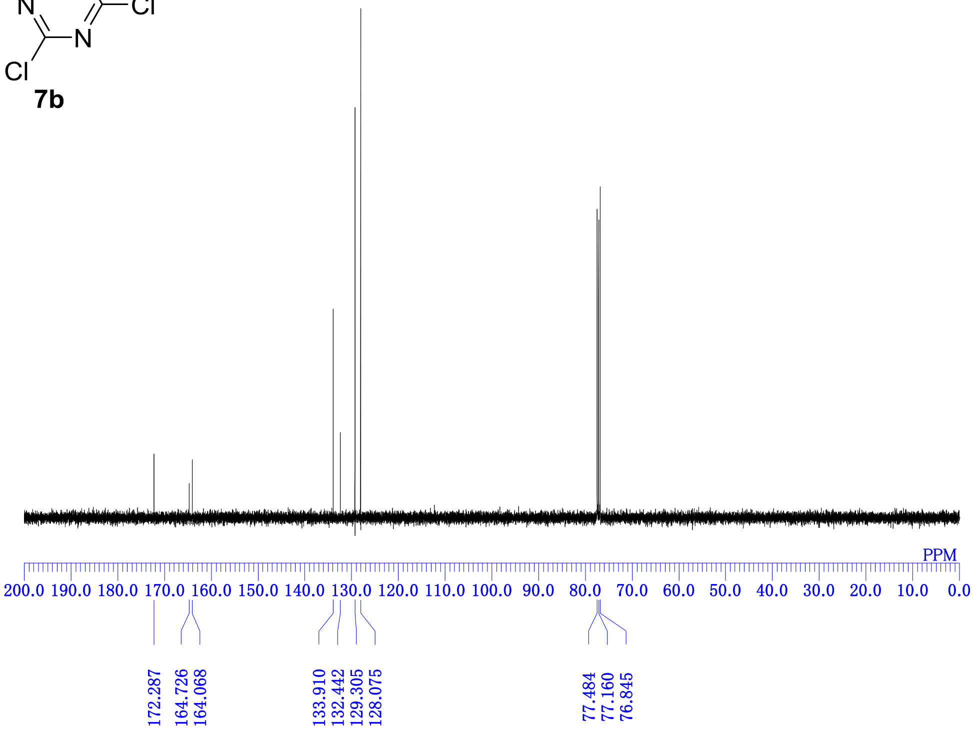
PPM
200.0 190.0 180.0 170.0 160.0 150.0 140.0 130.0 120.0 110.0 100.0 90.0 80.0 70.0 60.0 50.0 40.0 30.0 20.0 10.0 0.0
172.287
164.726
164.068
133.910
132.442
129.305
128.075
77.484
77.160
76.845

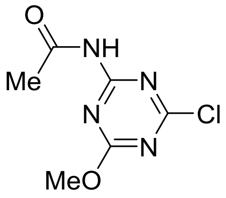
O
NH
Me
N
N
Cl
N
MeO

I

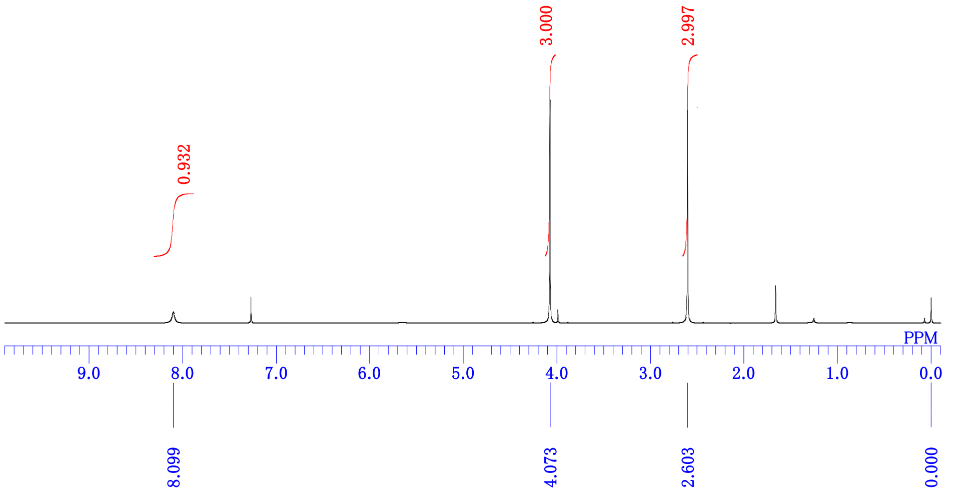
0.932
3.000
2.997
PPM
9.0 8.0 7.0 6.0 5.0 4.0 3.0 2.0 1.0 0.0
8.099
4.073
2.603
0.000

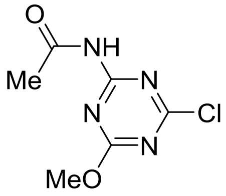
O
NH
Me
N
N
Cl
N
MeO

I

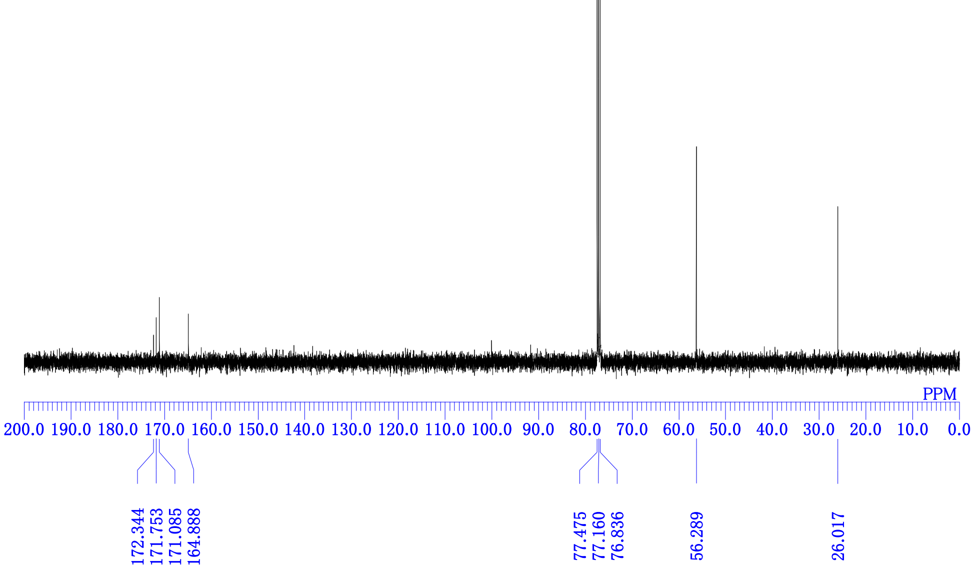
PPM
200.0 190.0 180.0 170.0 160.0 150.0 140.0 130.0 120.0 110.0 100.0 90.0 80.0 70.0 60.0 50.0 40.0 30.0 20.0 10.0 0.0
172.344
171.753
171.085
164.888
77.475
77.160
76.836
56.289
26.017

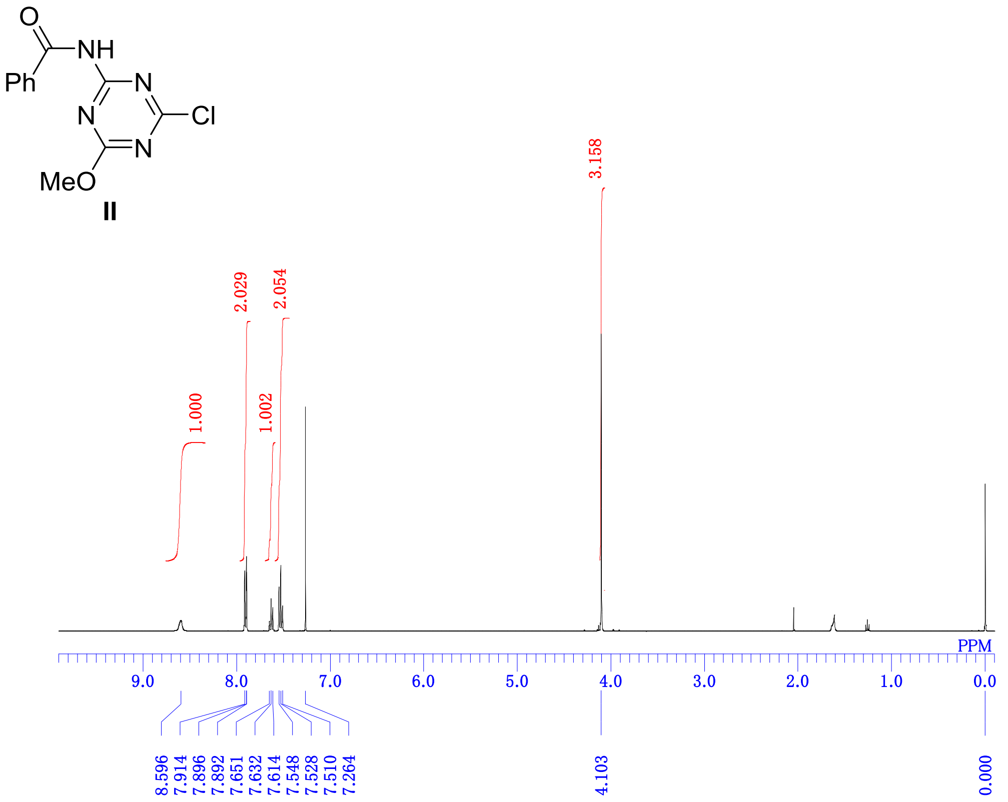

O
NH
Ph
N
N
Cl
N
MeO
II
3.158
2.029
2.054
1.000
1.002
PPM
9.0 8.0 7.0 6.0 5.0 4.0 3.0 2.0 1.0 0.0
8.596
7.914
7.896
7.892
7.651
7.632
7.614
7.548
7.528
7.510
7.264
4.103
0.000

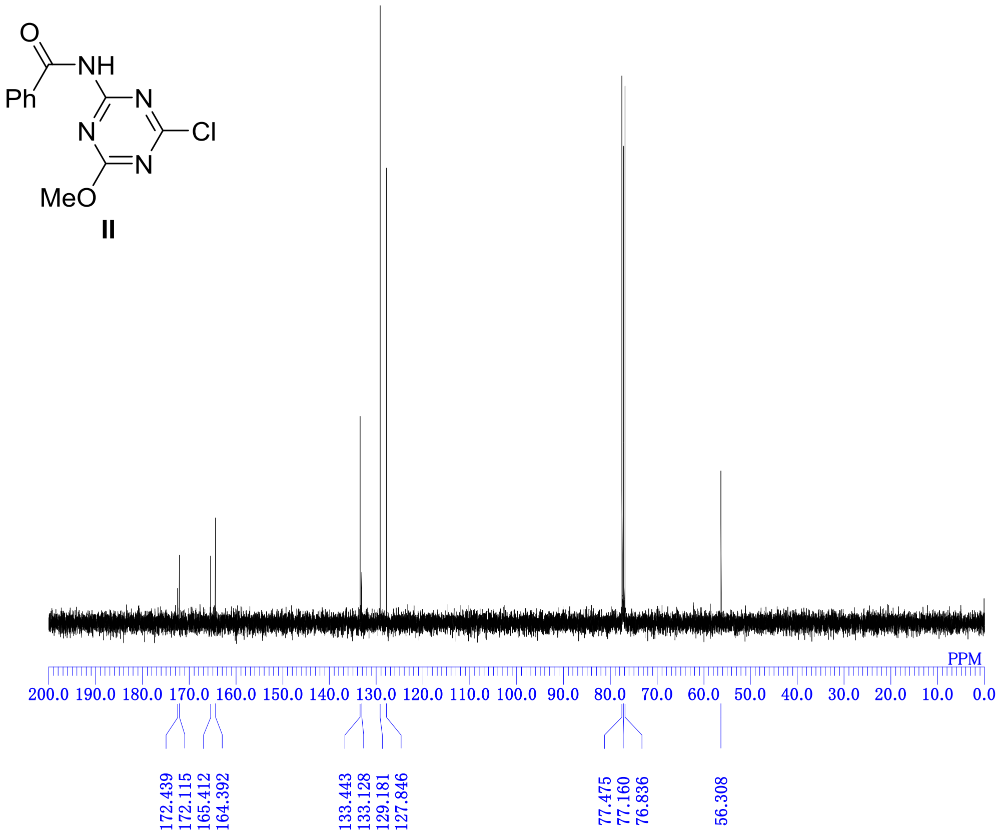

O
NH
Ph
N
N
Cl
N
MeO
II
PPM
200.0 190.0 180.0 170.0 160.0 150.0 140.0 130.0 120.0 110.0 100.0 90.0 80.0 70.0 60.0 50.0 40.0 30.0 20.0 10.0 0.0
172.439
172.115
165.412
164.392
133.443
133.128
129.181
127.846
77.475
77.160
76.836
56.308

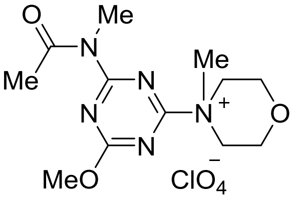
O
Me
N
Me
N
N
N
Me
N+
O
MeO
ClO4−

VII

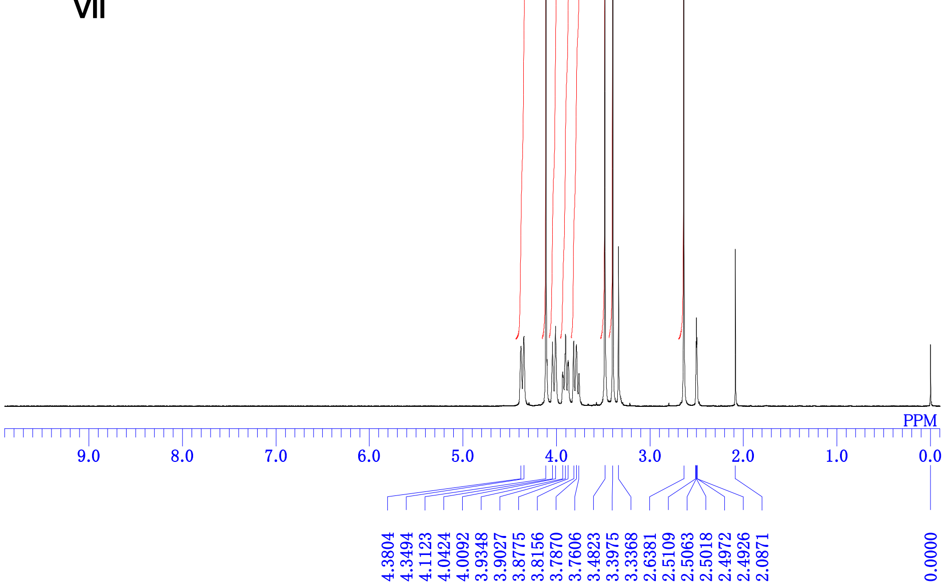
2.027
3.060
2.108
2.122
2.080
3.000
PPM
9.0
8.0
7.0
6.0
5.0
4.0
3.0
2.0
1.0
0.0
4.3804
4.3494
4.1123
4.0424
4.0092
3.9348
3.9027
3.8775
3.8156
3.7870
3.7606
3.4823
3.3975
3.3368
2.6381
2.5109
2.5063
2.5018
2.4972
2.4926
2.0871
0.0000

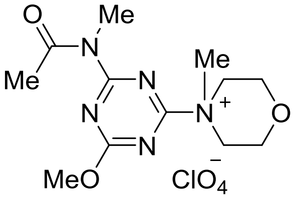
O
Me
N
Me
N
N
N
Me
N+
O
MeO
ClO4−

VII

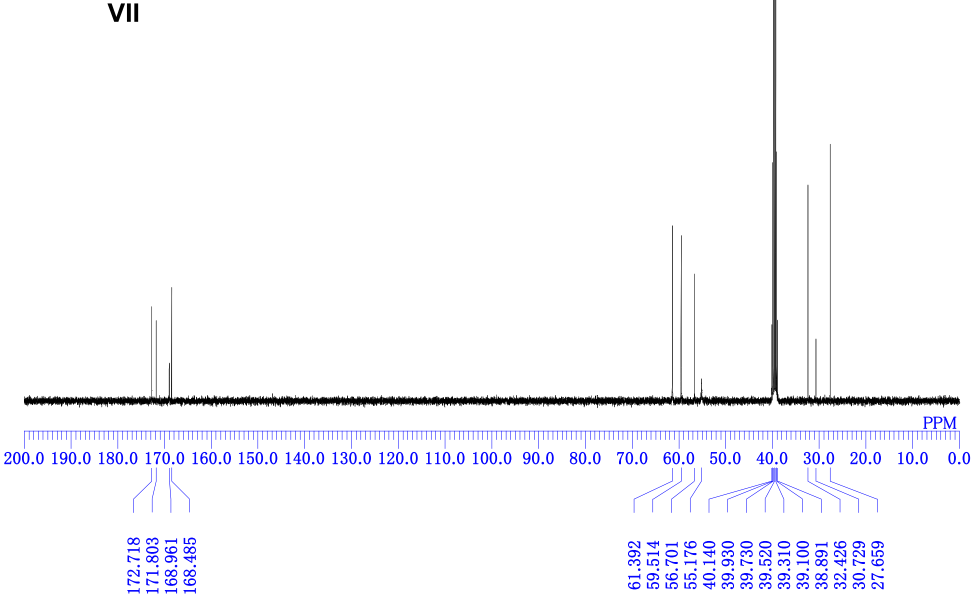
PPM
200.0
190.0
180.0
170.0
160.0
150.0
140.0
130.0
120.0
110.0
100.0
90.0
80.0
70.0
60.0
50.0
40.0
30.0
20.0
10.0
0.0
172.718
171.803
168.961
168.485
61.392
59.514
56.701
55.176
40.140
39.930
39.730
39.520
39.310
39.100
38.891
32.426
30.729
27.659

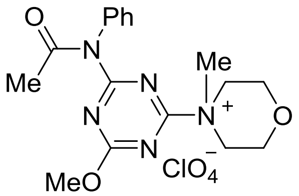
O
Ph
N
Me
N
Me
N
N+
O
N
ClO4−
MeO

VIII

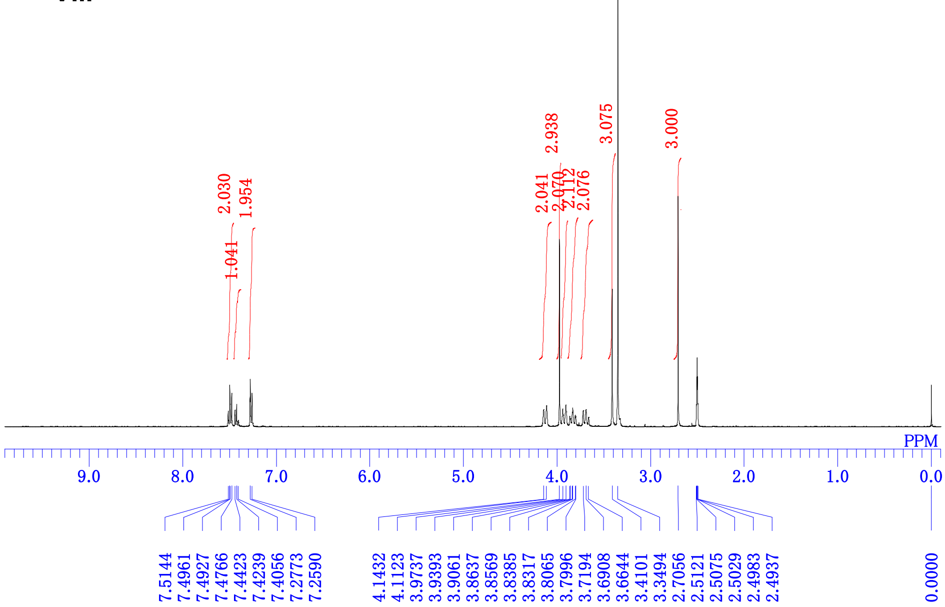
1.041
2.030
1.954
2.041
2.070
2.112
2.076
2.938
3.075
3.000
PPM
9.0 8.0 7.0 6.0 5.0 4.0 3.0 2.0 1.0 0.0
7.5144
7.4961
7.4927
7.4766
7.4423
7.4239
7.4056
7.2773
7.2590
4.1432
4.1123
3.9737
3.9393
3.9061
3.8637
3.8569
3.8385
3.8317
3.8065
3.7996
3.7194
3.6908
3.6644
3.4101
3.3494
2.7056
2.5121
2.5075
2.5029
2.4983
2.4937
0.0000

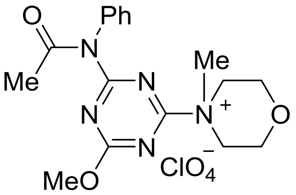
O
Ph
N
Me
N
Me
N
N+
O
N
ClO4−
MeO

VIII

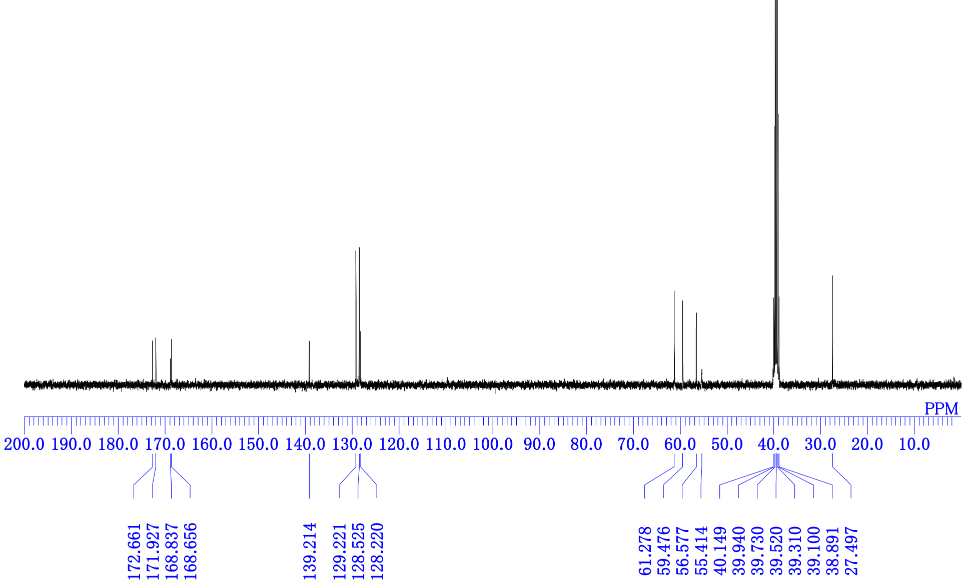
PPM
200.0 190.0 180.0 170.0 160.0 150.0 140.0 130.0 120.0 110.0 100.0 90.0 80.0 70.0 60.0 50.0 40.0 30.0 20.0 10.0
172.661
171.927
168.837
168.656
139.214
129.221
128.525
128.220
61.278
59.476
56.577
55.414
40.149
39.940
39.730
39.520
39.310
39.100
38.891
27.497

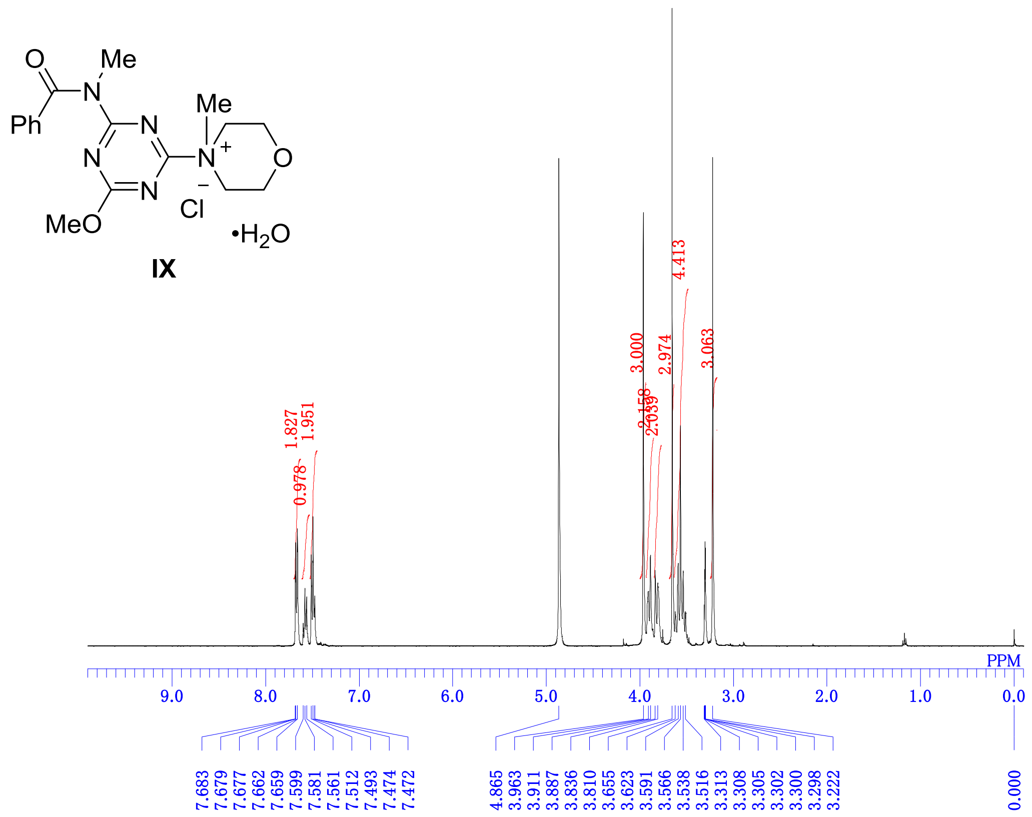
PPM
IX

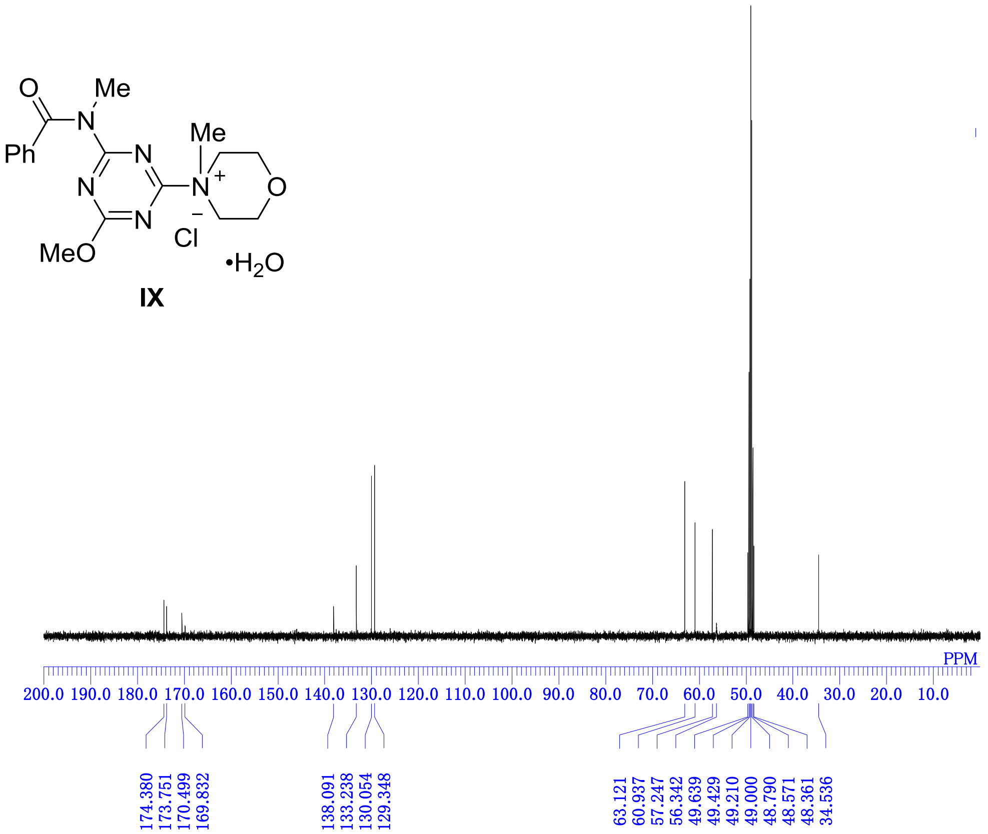
PPM
IX

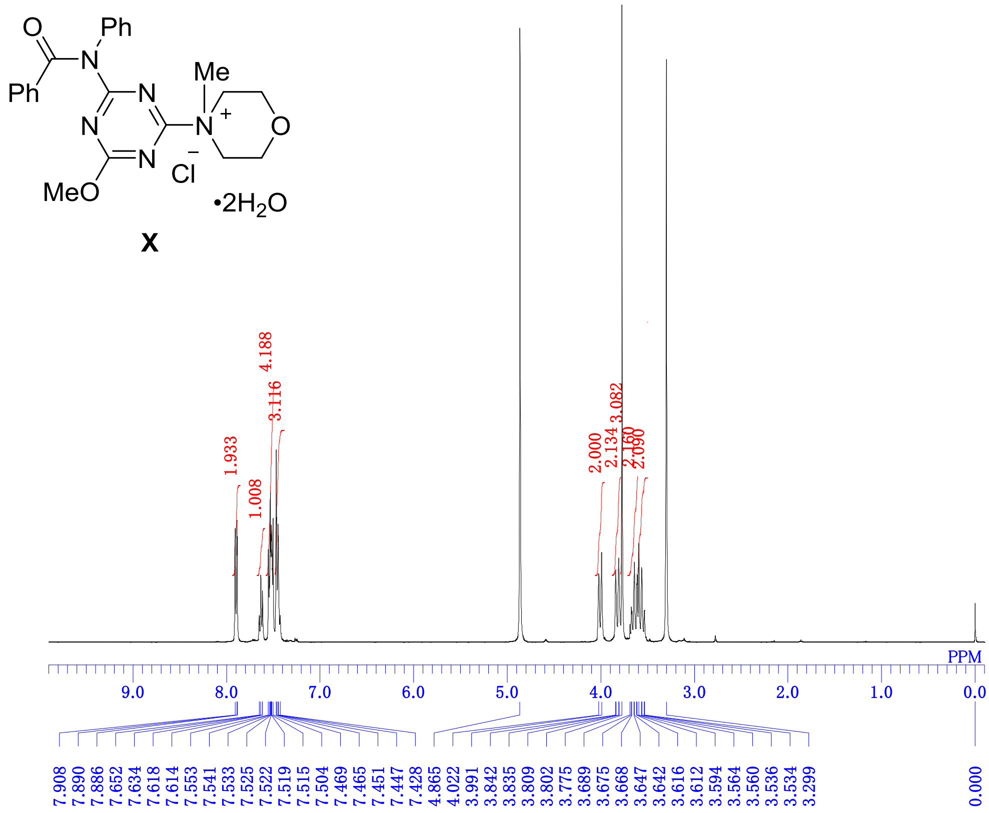

Ph
O
N
Ph
N
N
N
Me
N
+
O
MeO
Cl
–
•2H_2O
X
1.933
1.008
4.188
3.116
2.000
2.134
3.082
2.160
2.090
PPM
9.0 8.0 7.0 6.0 5.0 4.0 3.0 2.0 1.0 0.0
7.908 7.890 7.886 7.652 7.634 7.618 7.614 7.553 7.541 7.533 7.525 7.522 7.519 7.515 7.504 7.469 7.465 7.451 7.447 7.428 4.865 4.022 3.991 3.842 3.835 3.809 3.802 3.775 3.689 3.675 3.668 3.647 3.642 3.616 3.612 3.594 3.564 3.560 3.536 3.534 3.299
0.000

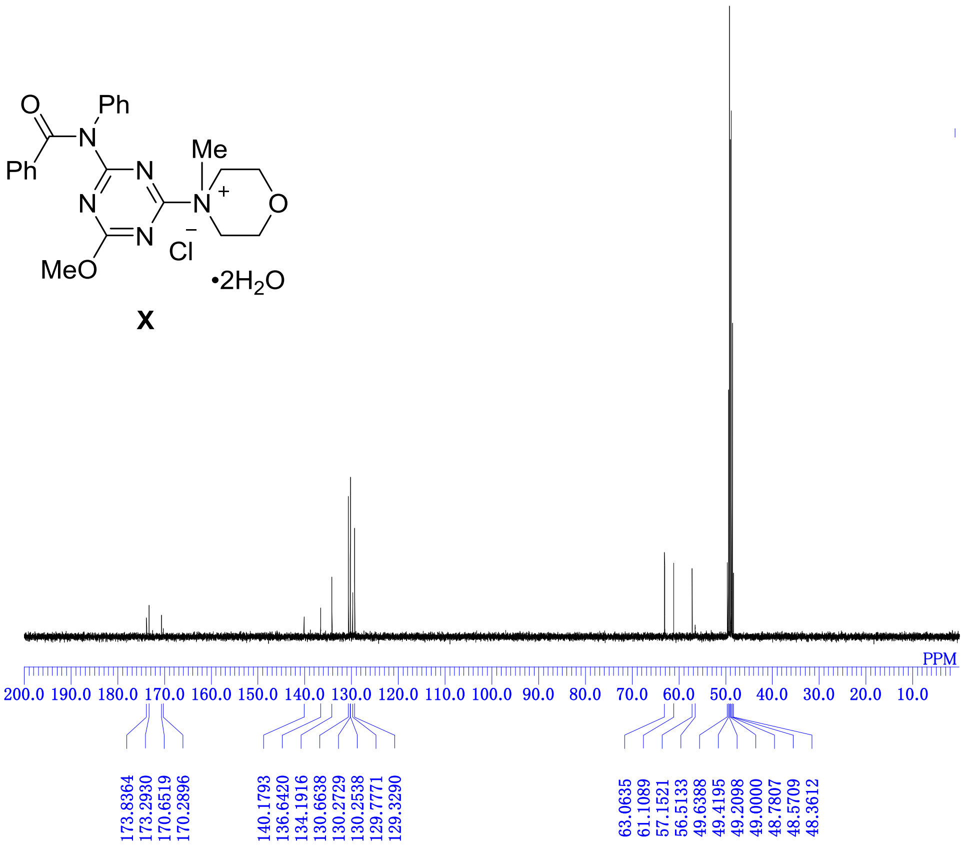

Ph
O
N
Ph
N
N
N
Me
N
+
O
MeO
Cl
–
•2H_2O
X
PPM
200.0 190.0 180.0 170.0 160.0 150.0 140.0 130.0 120.0 110.0 100.0 90.0 80.0 70.0 60.0 50.0 40.0 30.0 20.0 10.0
173.8364 173.2930 170.6519 170.2896
140.1793 136.6420 134.1916 130.6638 130.2729 130.2538 129.7771 129.3290
63.0635 61.1089 57.1521 56.5133 49.6388 49.4195 49.2098 49.0000 48.7807 48.5709 48.3612

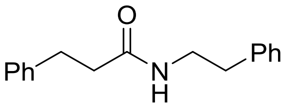

3a

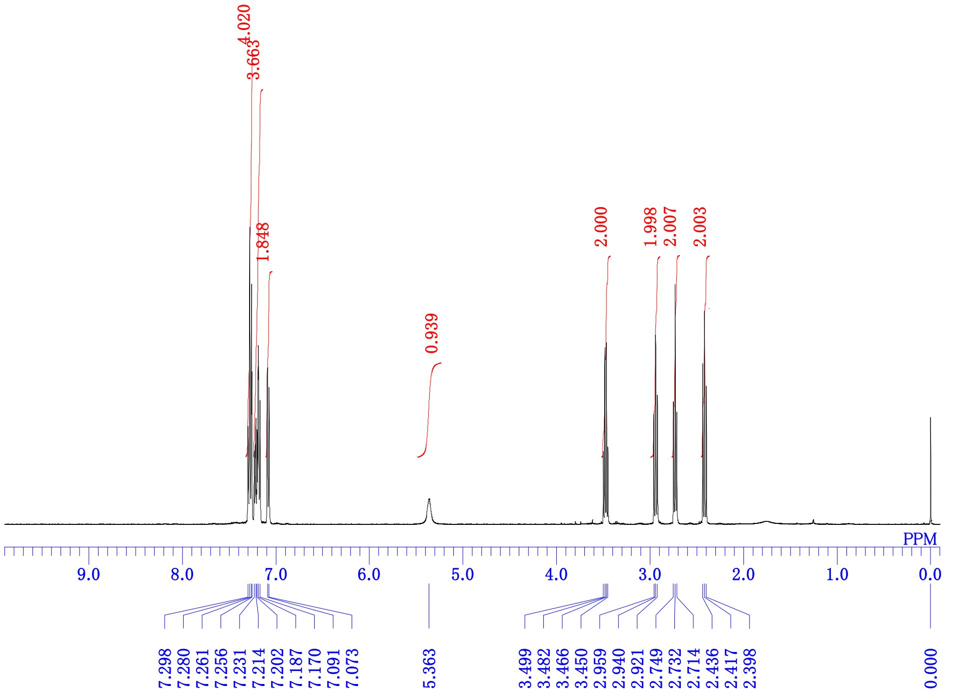

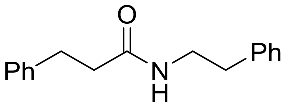

3a

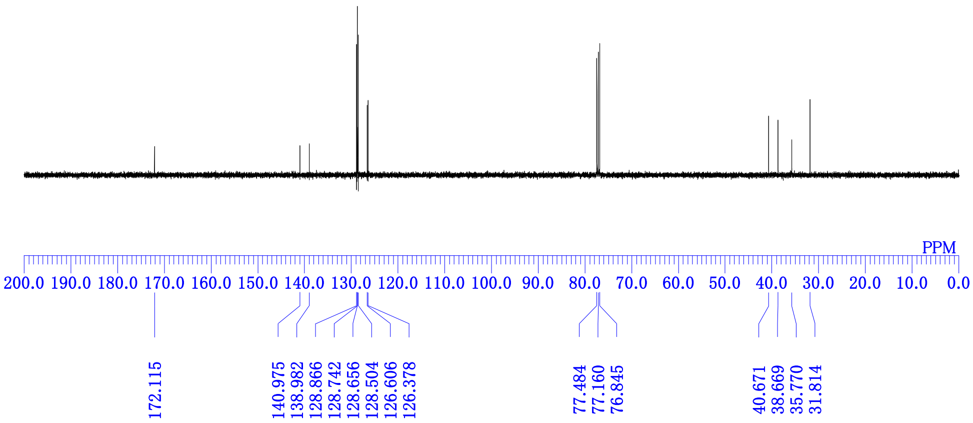

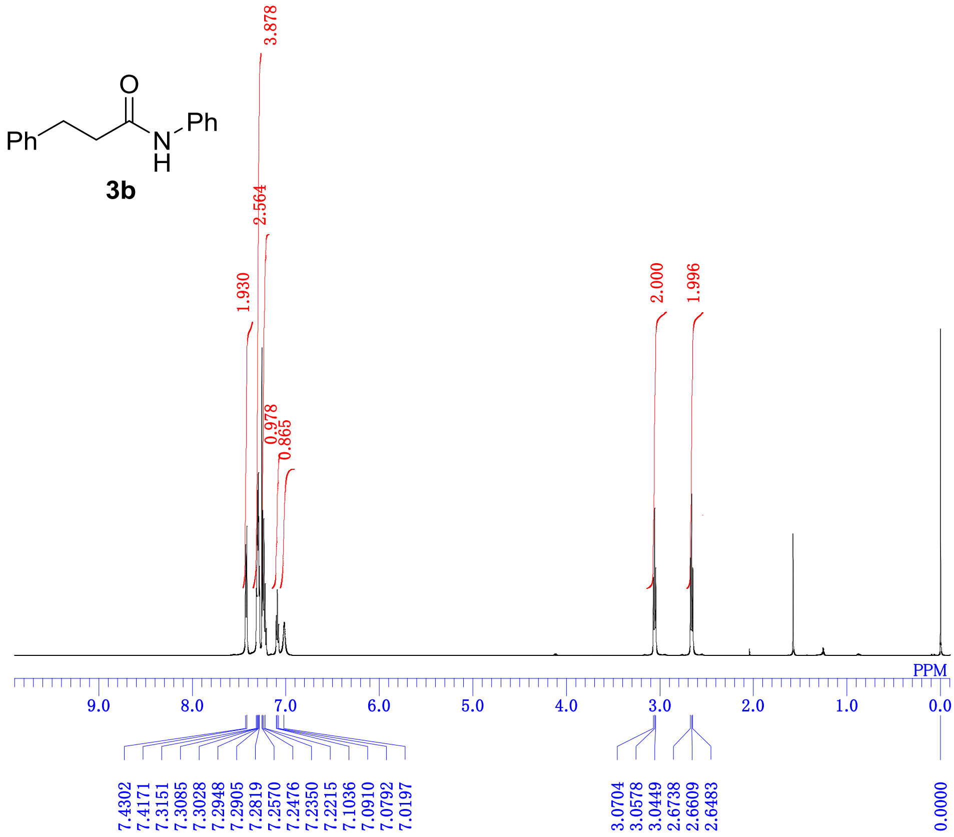
Ph
O
N
H
Ph
3b
3.878
2.564
1.930
0.978
0.865
2.000
1.996
PPM
9.0 8.0 7.0 6.0 5.0 4.0 3.0 2.0 1.0 0.0
7.4302
7.4171
7.3151
7.3085
7.3028
7.2948
7.2905
7.2819
7.2570
7.2476
7.2350
7.2215
7.1036
7.0910
7.0792
7.0197
3.0704
3.0578
3.0449
2.6738
2.6609
2.6483
0.0000

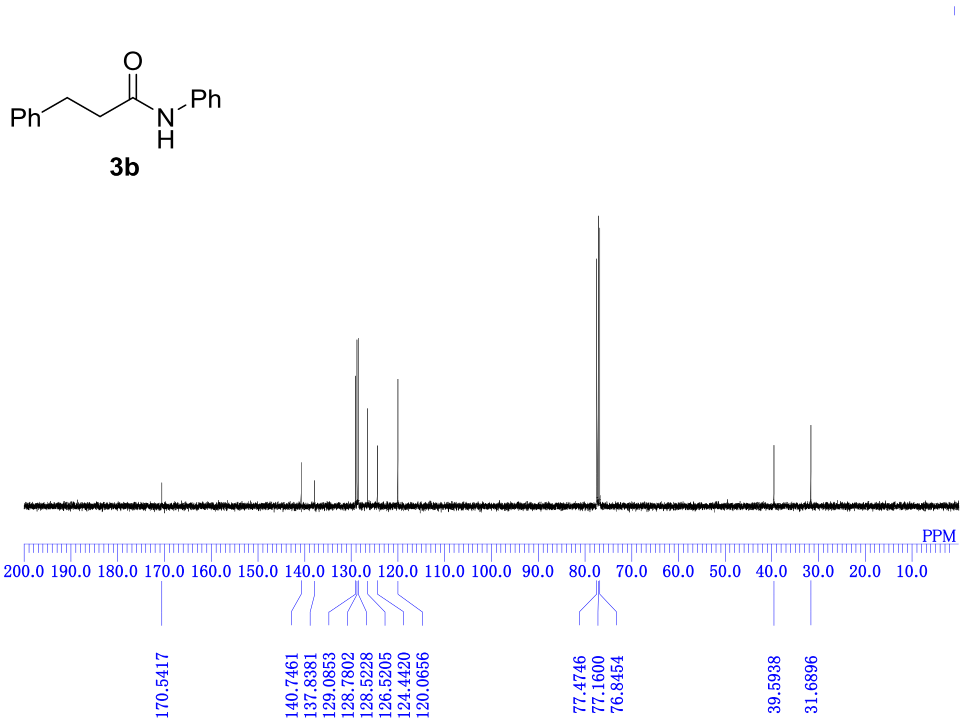
Ph
O
N
H
Ph
3b
PPM
200.0 190.0 180.0 170.0 160.0 150.0 140.0 130.0 120.0 110.0 100.0 90.0 80.0 70.0 60.0 50.0 40.0 30.0 20.0 10.0
170.5417
140.7461
137.8381
129.0853
128.7802
128.5228
126.5205
124.4420
120.0656
77.4746
77.1600
76.8454
39.5938
31.6896

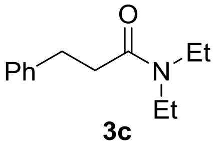
O
Ph
N
Et
Et

3c

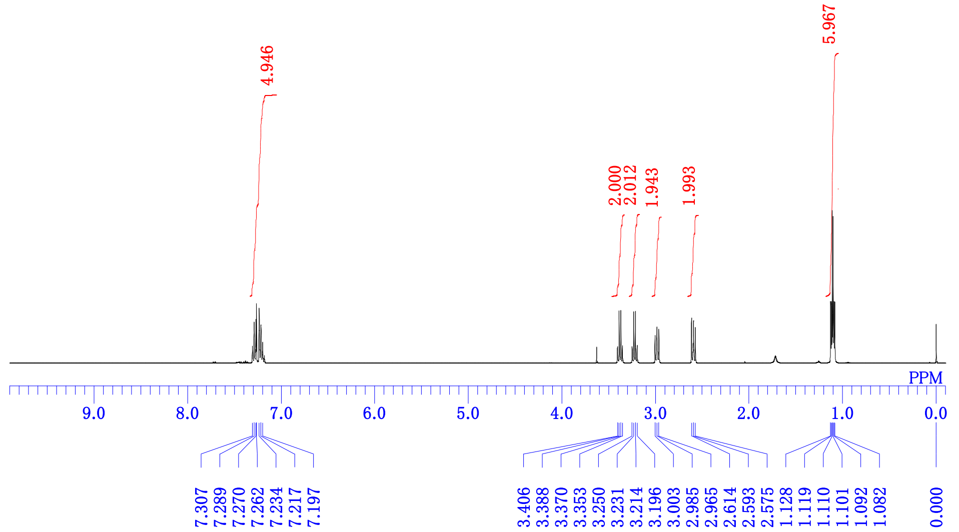
4.946
2.000
2.012
1.943
1.993
5.967
PPM
9.0 8.0 7.0 6.0 5.0 4.0 3.0 2.0 1.0 0.0
7.307 7.289 7.270 7.262 7.234 7.217 7.197
3.406 3.388 3.370 3.353 3.250 3.231 3.214 3.196 3.003 2.985 2.965 2.614 2.593 2.575 1.128 1.119 1.110 1.101 1.092 1.082
0.000

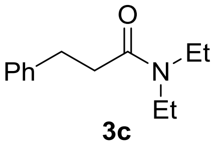
O
Ph
N
Et
Et

3c

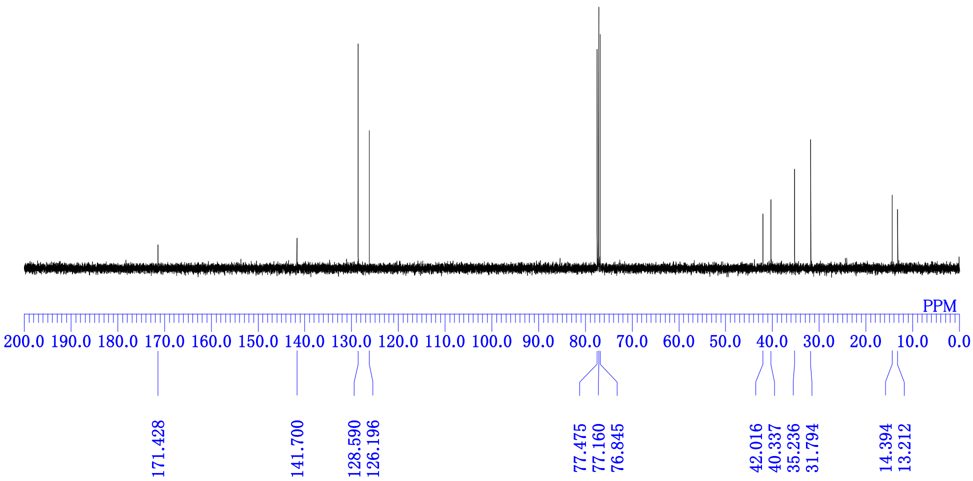
PPM
200.0 190.0 180.0 170.0 160.0 150.0 140.0 130.0 120.0 110.0 100.0 90.0 80.0 70.0 60.0 50.0 40.0 30.0 20.0 10.0 0.0
171.428
141.700
128.590 126.196
77.475 77.160 76.845
42.016 40.337 35.236 31.794
14.394 13.212

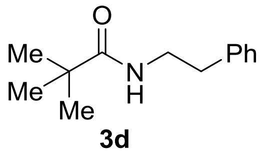
Me
Me
Me
O
N
H
Ph
3d

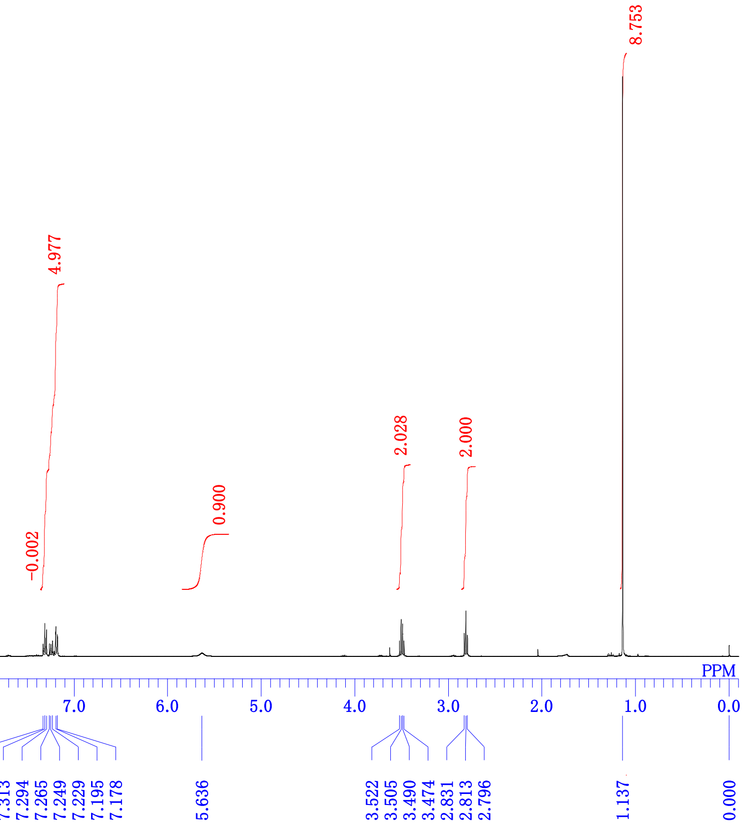
8.753
4.977
-0.002
0.900
2.028
2.000
PPM
9.0 8.0 7.0 6.0 5.0 4.0 3.0 2.0 1.0 0.0
7.331 7.313 7.294 7.265 7.249 7.229 7.195 7.178
5.636
3.522 3.505 3.490 3.474 2.831 2.813 2.796
1.137
0.000

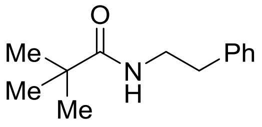
Me
Me
Me
O
N
H
Ph
3d

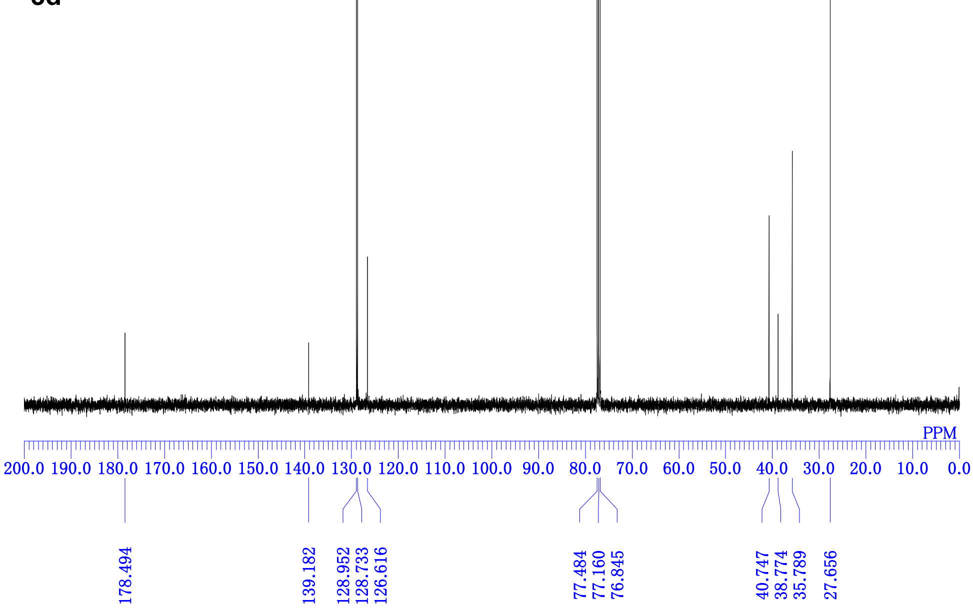
PPM
200.0 190.0 180.0 170.0 160.0 150.0 140.0 130.0 120.0 110.0 100.0 90.0 80.0 70.0 60.0 50.0 40.0 30.0 20.0 10.0 0.0
178.494
139.182
128.952 128.733 126.616
77.484 77.160 76.845
40.747 38.774 35.789
27.656

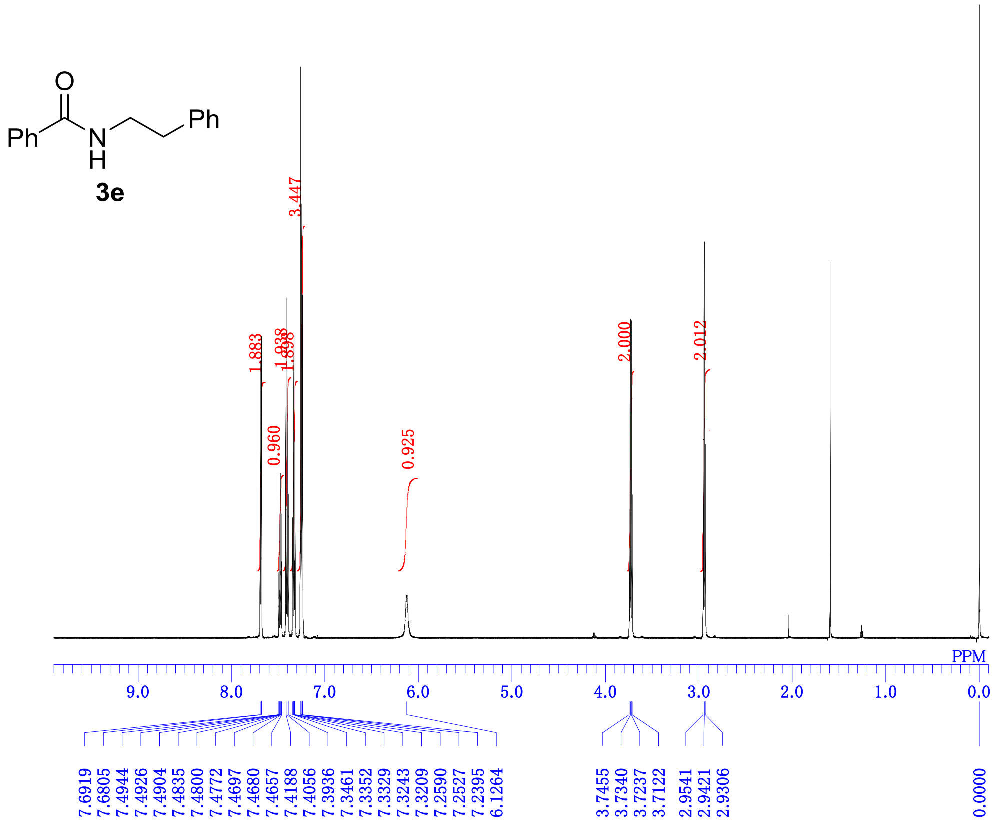

Ph
N
H
Ph
O
3e

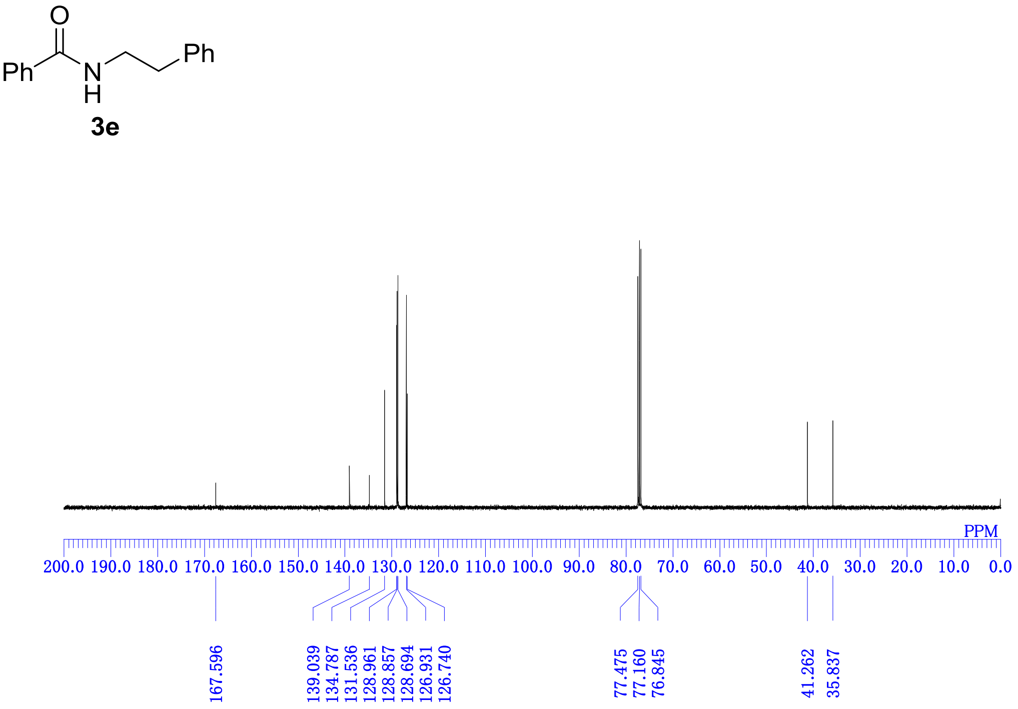

Ph
N
H
Ph
O
3e

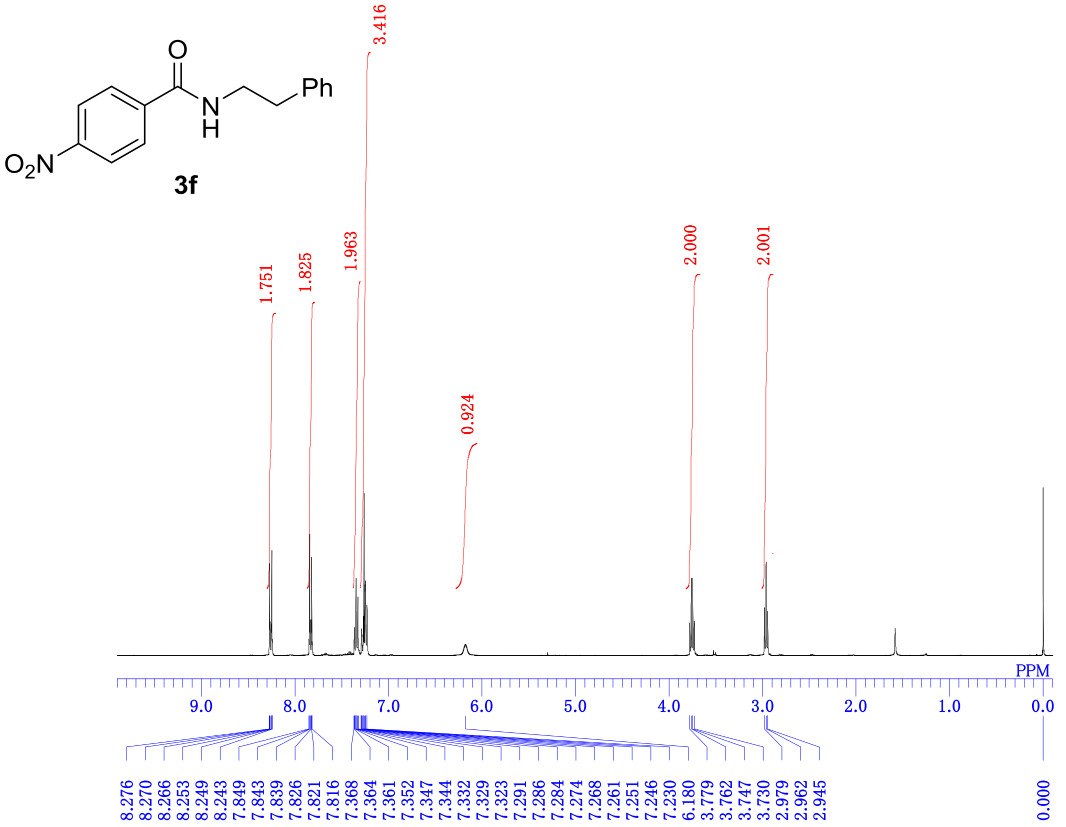

O
N
H
Ph
O_2N
3f
1.751
1.825
1.963
3.416
0.924
2.000
2.001
PPM
9.0
8.0
7.0
6.0
5.0
4.0
3.0
2.0
1.0
0.0
8.276 8.270 8.266 8.253 8.249 8.243 7.849 7.843 7.839 7.826 7.821 7.816 7.368 7.364 7.361 7.352 7.347 7.344 7.332 7.329 7.323 7.291 7.286 7.284 7.274 7.268 7.261 7.251 7.246 7.230 6.180 3.779 3.762 3.747 3.730 2.979 2.962 2.945
0.000

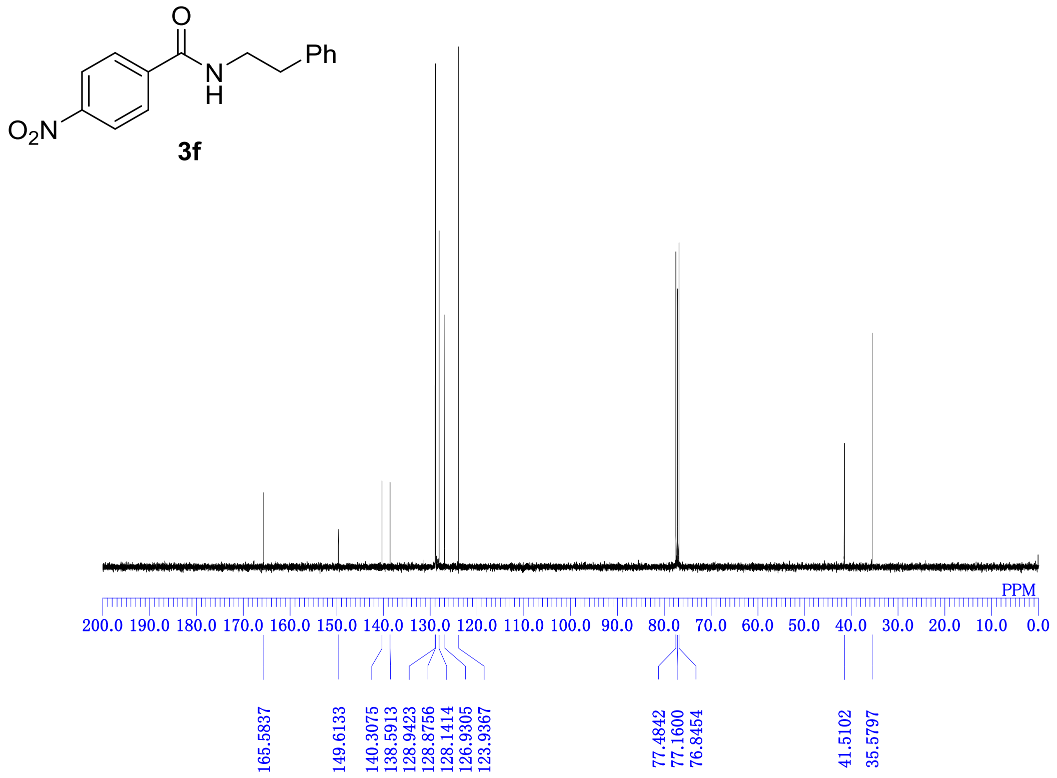

O
N
H
Ph
O_2N
3f
PPM
200.0 190.0 180.0 170.0 160.0 150.0 140.0 130.0 120.0 110.0 100.0 90.0 80.0 70.0 60.0 50.0 40.0 30.0 20.0 10.0 0.0
165.5837
149.6133
140.3075 138.5913 128.9423 128.8756 128.1414 126.9305 123.9367
77.4842 77.1600 76.8454
41.5102 35.5797

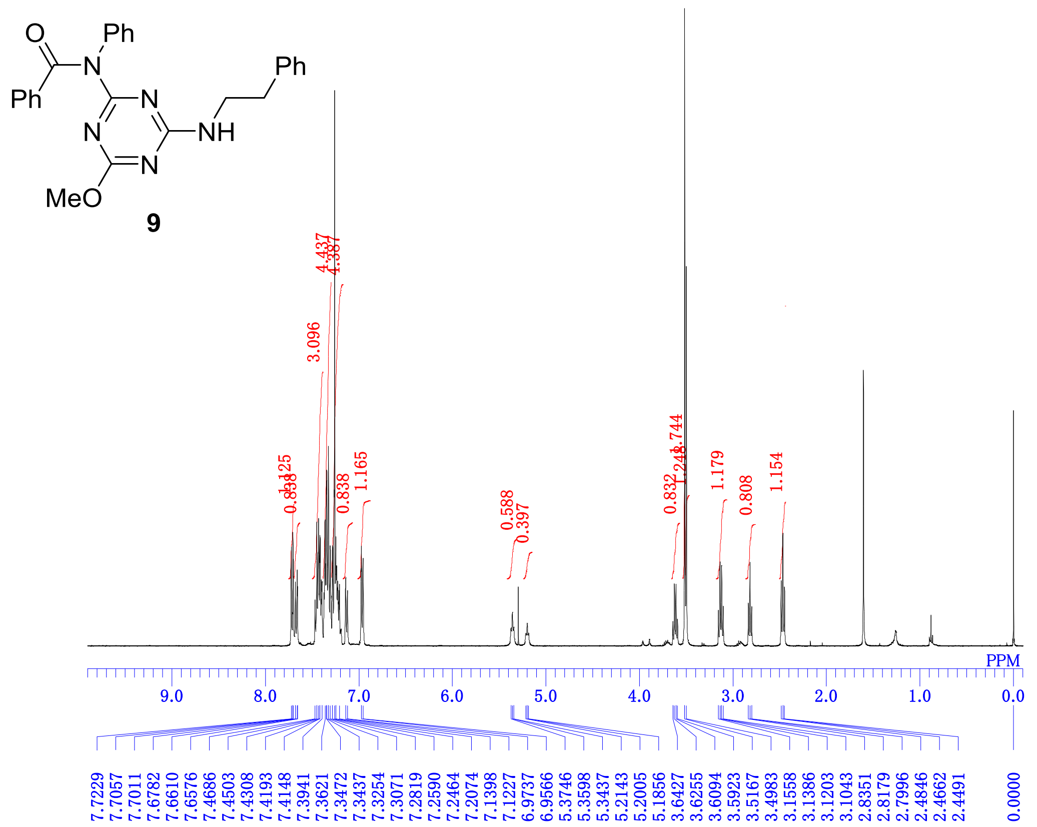

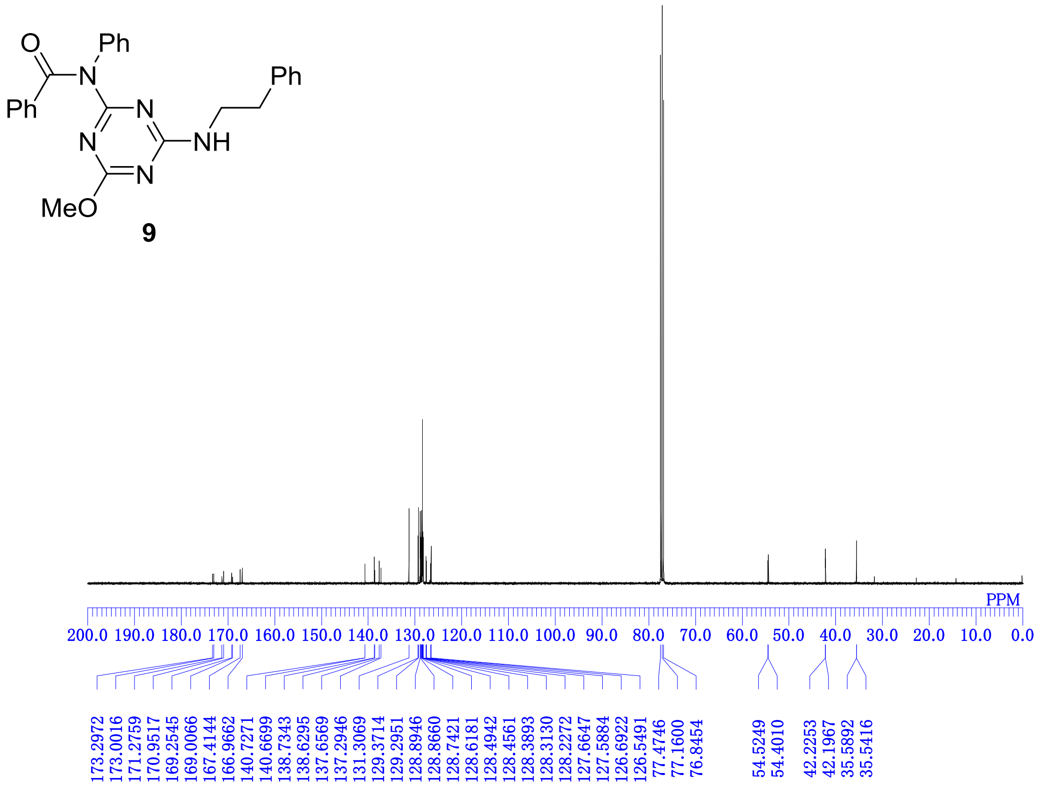

References

1. Kunishima, M.; Kawachi, C.; Morita, J.; Terao, K.; Iwasaki, F.; Tani, S. *Tetrahedron*, **1999**, *55*, 13159–13170.
2. Sheikh, M. C.; Takagi, S.; Yoshimura, T.; Morita H. *Tetrahedron*, **2010**, *66*, 7272–7278.
3. Shiina, I.; Kawakita, Y. *Tetrahedron,* **2004**, *60*, 4729–4733.
4. Funasaka, S.; Mukaiyama, T. *Bull. Chem. Soc. Jpn.*, **2008**, *81*, 148–159.
5. Chen, P.-J.; Wang, H.-Y,; Peng, A.-Y. *RSC Adv.*, **2015**, *5*, 94328–94331.
6. Boehner, C. M.; Marsden, D. M.; Sore, H. F.; Norton, D.; Spring, D. R. *Tetrahedron, Lett.*, **2010**, *51*, 5930–5932.
7. Lenstra, D. C.; Rutjes, F. P. J. T.; Mecinović, J. *Chem. Commun.*, **2014**, *50*, 5763–5766.
